# Material‐Encoded Synchronization of Immunogenic Cell Death With Adenosine A2A Receptor Blockade Reprograms the Tumor Microenvironment

**DOI:** 10.1002/advs.76303

**Published:** 2026-06-28

**Authors:** Xiangting Yi, Hanlou Yang, Junting Huang, Pengfei Wu, Shiying Zhou, Zunde Liao, Min Han, Zishan Chen, Xiaoyu Huang, Nan Ma, Jie Zhou, Mingqiang Li, Yiling Zhong

**Affiliations:** ^1^ State Key Laboratory of Bioactive Molecules and Druggability Assessment Guangdong Basic Research Center of Excellence for Natural Bioactive Molecules and Discovery of Innovative Drugs College of Pharmacy Jinan University Guangzhou Guangdong P. R. China; ^2^ Department of Breast Surgery Affiliated Cancer Hospital and Institute of Guangzhou Medical University Guangzhou P. R. China; ^3^ Laboratory of Biomaterials and Translational Medicine Center for Nanomedicine The Third Affiliated Hospital Sun Yat‐sen University Guangzhou P. R. China

**Keywords:** adenosine, adenosine 2A receptor, immunogenic cell death, nanosystem, tumor microenvironment reprogramming

## Abstract

Adenosine rapidly suppresses antitumor immunity through the adenosine A2A receptor (A2AR). Immunogenic cell death (ICD) releases extracellular ATP, which can be rapidly converted into immunosuppressive adenosine. We therefore designed BSCS@PHY to synchronize ICD induction with local A2AR blockade in the same spatiotemporal window, aiming to protect antigen priming from adenosine‐mediated suppression. BSCS@PHY integrates a bismuth–copper diselenide core for photothermal heating, glutathione depletion, and chemodynamic hydroxyl‐radical generation; hyaluronic‐acid‐modified phase‐change materials for on‐site activation; the A2AR antagonist SCH442416 for local A2AR blockade; and yeast cell wall (YCW) components for innate adjuvanticity. Thermography‐guided irradiation confines activation within a controlled window, melts the phase‐change shell to expose the catalytic surface, and coordinates ICD amplification with co‐localized A2AR blockade and dendritic‐cell activation. In 4T1 tumors, BSCS@PHY enables image‐guided activation, enhances ICD hallmarks, lowers adenosine signaling, promotes dendritic‐cell maturation and T‐cell priming, and improves tumor control, with additional benefit when combined with anti‐PD‐L1. Loss‐of‐function comparisons support nonredundant contributions of A2AR blockade and YCW‐mediated adjuvanticity. This material‐encoded strategy aligns danger‐signal generation with local A2AR blockade in the same tumor niche and offers a framework for pairing ICD induction with metabolic‐checkpoint control in cold tumors.

## Graphical abstract

A bismuth–copper diselenide–based nanoplatform (BSCS@PHY) coordinates immunogenic cell death with local A2A receptor blockade in 4T1 tumors. Thermally triggered shell melting exposes catalytic surfaces for glutathione depletion and chemodynamic ROS generation, while co‐delivering an A2AR antagonist and yeast‐wall adjuvant to enhance dendritic‐cell activation, T‐cell priming, and anti‐PD‐L1–responsive tumor control.

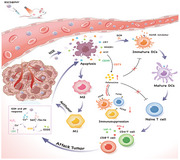

## 1 Introduction

The tumor microenvironment (TME) couples metabolic and immunologic constraints that blunt antitumor immunity [1, 2]. Among them, adenosine signaling has emerged as a central suppressive axis: extracellular ATP released during cellular stress or death is swiftly hydrolyzed by CD39/CD73 to adenosine [3], which engages the adenosine A2A receptor (A2AR) expressed by T cells, dendritic cells (DCs), and natural killer (NK) cells to attenuate effector function, favor regulatory lineages, and facilitate tumor progression [4, 5, 6]. Pharmacologic A2AR blockade can revive cytotoxicity, improve cross‐presentation, and diminish the accumulation of suppressive myeloid cells and regulatory T cells (Tregs), thereby providing a rationale for A2AR‐centered combination therapies to reprogram the TME [2, 7, 8, 9, 10]. Because ICD‐derived ATP can be rapidly converted to adenosine, A2AR‐centered strategies need to be aligned with this short‐lived immunologic window. However, most ICD‐based nanoplatforms are designed primarily to amplify danger‐signal release and tumor antigen exposure, while insufficiently addressing the local ATP‐to‐adenosine transition that can impose A2AR‐mediated suppression at the same site and time [6, 11, 12]. This spatiotemporal mismatch provides the rationale for a material system that can synchronize ICD induction with local A2AR blockade.

In particular, ICD‐inducing modalities such as photothermal therapy (PTT) and chemodynamic therapy (CDT) induce calreticulin (CRT) exposure, release other damage‐associated molecular patterns (DAMPs), and liberate tumor antigens, thereby promoting innate immune activation and subsequent adaptive T‐cell priming [13, 14, 15]. However, the same ICD cascade also increases extracellular ATP, which can be rapidly converted to adenosine, thereby enabling A2AR‐mediated suppression of antigen presentation and T‐cell priming [10, 16, 17]. Because extracellular adenosine can arise through both CD39/CD73‐dependent and alternative enzymatic routes, targeting A2AR provides a downstream strategy to intercept this suppressive signal with less vulnerability to compensation by upstream adenosine‐generating pathways [6, 18, 19, 20, 21, 22]. Given the transient nature of the adenosine surge after ICD [6, 23], local A2AR blockade should be synchronized with ICD‐derived danger‐signal release, thereby preserving immune priming while avoiding broad disruption of the CD39/CD73‐associated homeostatic axis [24, 25].

Nanomaterials are well‐suited to encode such spatiotemporally staged interventions [26]. Bismuth nanostructures provide strong near‐infrared absorbance and efficient photothermal conversion [27]; copper centers catalyze Fenton‐like reactions to generate hydroxyl radicals for CDT under tumor‐relevant conditions [15]; and selenium chemistry, through labile diselenide bonds, enables glutathione (GSH)‐responsive behavior that both depletes intratumoral GSH and potentiates redox‐based cytotoxicity [28, 29, 30]. Hyaluronic acid (HA) coatings support biocompatibility and CD44‐mediated tumor targeting [31, 32], while phase‐change materials (PCMs) confer thermally gated release near the melting point [33, 34]. In parallel, yeast‐cell‐wall β‐glucans act as pathogen‐associated molecular patterns that engage dectin‐1 and related receptors on antigen‐presenting cells (APCs), thereby providing innate adjuvanticity to promote dendritic‐cell maturation and subsequent T‐cell priming [35, 36].

**FIGURE 1:**
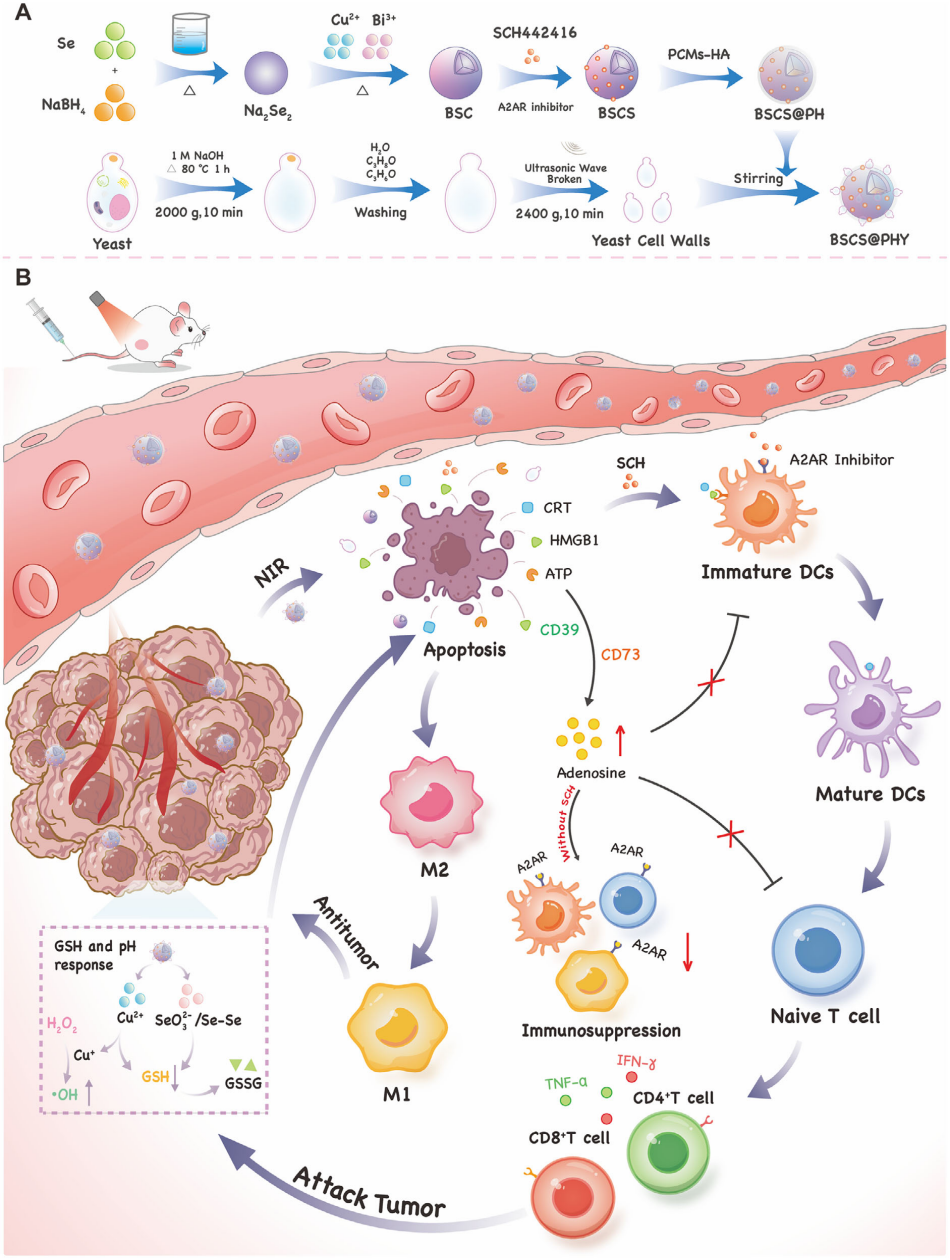
Synthesis and therapeutic mechanisms of BSCS@PHY nanosystem. (A) Schematic representation of the preparation of BSCS@PHY nanosystem. (B) Schematic of antitumor immunity induced by BSCS@PHY. Upon NIR laser irradiation, BSCS@PHY produces photothermal ablation and melts the PCM shell to expose the BSC core; in the GSH rich TME, diselenide bonds are cleaved, depleting GSH and releasing Cu ions that catalyze hydroxyl radical generation (CDT), collectively triggering ICD with the release of tumor‐associated antigens (TAAs) and DAMPs. SCH442416 (SCH) blocks A2AR‐mediated immunosuppression, while yeast cell walls rich in β‐glucan promote DC activation and adaptive T‐cell responses.

Guided by this principle, we engineered BSCS@PHY, a thermography‐guided, staged‐cascade nanosystem that integrates the following components (Figure 1): (i) a bismuth‐copper diselenide (BSC) core to couple PTT with GSH depletion and CDT; (ii) HA‐modified phase‐change materials (PCMs‐HA) that remain stable below their melting point and undergo thermal transition under near‐infrared heating to expose the catalytic BSC surface and associated therapeutic components; (iii) the small‐molecule A2AR antagonist SCH442416 [37, 38] to counter adenosine‐mediated suppression during the window in which ICD‐derived ATP can be converted to adenosine; and (iv) yeast cell wall (YCW) to provide innate immune stimulation and promote APC maturation and cross‐priming. Real‐time thermography guides energy deposition to melt the PCM shell, unveiling the BSC surface for GSH‐consuming diselenide cleavage and Cu‐mediated hydroxyl‐radical generation, thereby reinforcing ICD. In the same spatiotemporal window, SCH442416 relieves A2AR signaling, while YCW provides pattern‐recognition cues that drive DC maturation. Thus, BSCS@PHY goes beyond an ICD‐only nanoplatform by functioning as a material‐encoded synchronization system that couples ICD induction, local A2AR blockade, and YCW‐mediated innate immune stimulation within the same tumor‐localized activation window.

These features distinguish BSCS@PHY from ICD‐only nanoplatforms and asynchronous combinations with systemic A2AR antagonists: co‐localized action in tumor‐localized immune niches, synchronized local A2AR blockade and YCW‐mediated innate immune stimulation during ICD induction, and thermography‐guided activation within a controlled temperature window. We therefore hypothesize that BSCS@PHY will (1) achieve on‐demand, image‐guided activation with spatiotemporally controlled therapeutic exposure; (2) deplete intratumoral GSH and enhance CDT to amplify ICD; (3) concomitantly block A2AR to preserve antigen presentation and effector T‐cell priming; and (4) leverage YCW to promote APC activation and immune priming—thereby converting “cold” tumors into immune‐responsive phenotypes and providing a rationale for combination with immune checkpoint blockade. This study details the design, physicochemical characterization, and mechanism‐centric evaluation of BSCS@PHY, and demonstrates how staged integration of PTT/CDT, metabolic‐checkpoint inhibition, and innate adjuvanticity can reprogram the TME for durable antitumor immunity.

## 2 Results and Discussion

### 2.1 Synthesis and Characterization of BSCS@PHY

**FIGURE 2:**
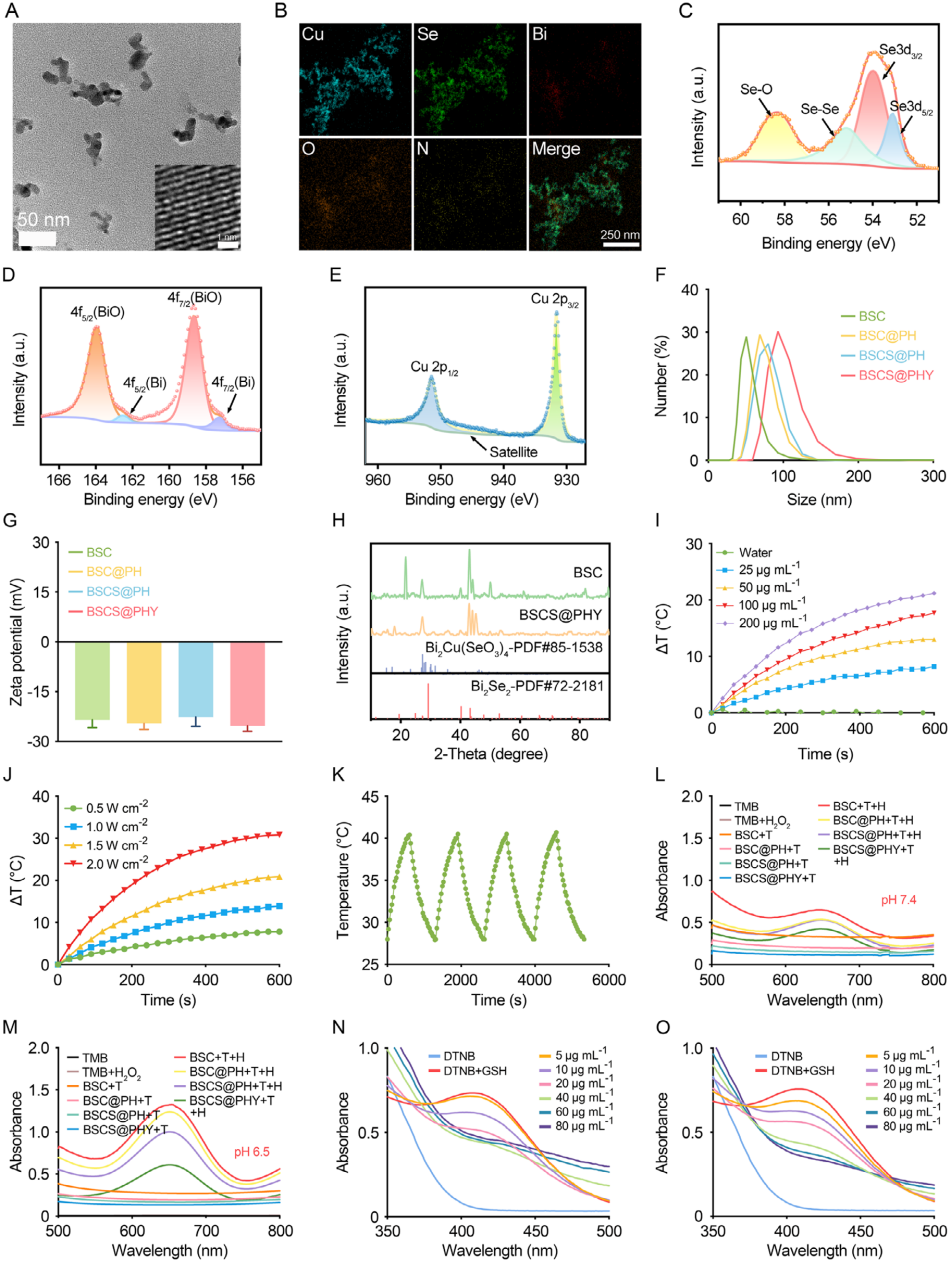
Characterizations of BSC and BSCS@PHY. (A) TEM and HRTEM images of BSC. (B) Elemental composition maps for BSC. (C‐E) High‐resolution Se 3d (C), Bi 4f (D), and Cu 2p (E) XPS spectra of BSC. (F, G) Hydrodynamic diameter (F) and Zeta potential (G) of various samples. (H) XRD pattern of BSC and BSCS@PHY. (I) Photothermal heating curves of BSC aqueous dispersions at graded concentrations under NIR irradiation (1 W cm^−^^2^, 10 min). (J) Temperature‐time profiles demonstrating the photothermal response of BSC at a concentration of 100 µg mL^−1^ in aqueous solution, subjected to NIR laser irradiation at various power densities. (K) Photothermal stability of BSC in aqueous solution, as shown by real‐time temperature curves during four on/off cycles of NIR laser exposure. Evaluation of ROS production. (L, M) UV‐vis absorption spectra depicting the reaction of TMB alone, TMB with H_2_O_2_, TMB with BSC‐based formulations, and TMB with both H_2_O_2_ and BSC‐based formulations, measured at pH 7.4 (L) and pH 6.5 (M) over 3 h at room temperature. (N) GSH depletion by BSC at the indicated concentrations, quantified with DTNB. (O) GSH depletion by BSCS@PHY at the indicated concentrations, quantified with DTNB.

A straightforward one‐pot method was employed to synthesize BSC (Figure 1A). Transmission electron microscopy (TEM) showed irregularly shaped BSC nanoparticles with a mean diameter of 21 ± 2.5 nm (Figure 2A). High‐resolution TEM (HRTEM) resolved clear lattice fringes, consistent with a crystalline BSC phase. Energy‐dispersive X‐ray spectroscopy (EDS) mapping verified the presence of copper (Cu), selenium (Se), bismuth (Bi), oxygen (O), and nitrogen (N) elements, consistent with the intended composition (Figure 2B). X‐ray photoelectron spectroscopy (XPS) was performed to assess the surface composition and oxidation states of BSC nanoparticles. The survey spectrum confirmed Cu, Se, Bi, O, and N (Figure S1). In the Se 3d region (Figure 2C), a doublet at ∼53.1/54.0 eV (3d_5/2_ / 3d_3/2_) is characteristic of lattice selenide (Se^2−^), accompanied by a component at ∼55.3 eV indicative of elemental Se/Se‐Se species, and a Se─O contribution at ∼58‐60 eV consistent with selenite/selenate from surface oxidation [39, 40, 41]. The Bi 4f spectrum (Figure 2D) shows a doublet at 157.3/162.6 eV typical of Bi^3+^ in bismuth selenide, together with a higher‐binding‐energy pair at 158.7/164.0 eV attributable to oxidized bismuth species (e.g., Bi_2_O_3_ or bismuth selenites/selenates) [42, 43]. The Cu 2p spectrum (Figure 2E) displays peaks at ∼931.6 eV (2p_3/2_) and ∼951.5 eV (2p_1/2_); the binding energies and weak satellite features are consistent with copper in a selenide environment (predominantly Cu(I) with possible Cu(II) contributions) [44, 45]. Collectively, these data confirm the formation of a bismuth‐copper selenide lattice with a thin, air‐exposed oxidized surface layer.

The UV‐vis‐NIR spectrum of BSC showed a broad band extending into the NIR region (Figure S2), consistent with strong light absorption for photothermal conversion. To block adenosine signaling at the receptor level, the A2AR antagonist SCH442416 was loaded into BSC to obtain BSCS. BSCS was then coated with PCMs‐HA to yield BSCS@PH, providing an HA interface and thermos‐responsive gating of payload release. The UV‐vis‐NIR spectrum of BSCS@PH retained the BSC NIR band and displayed additional features attributable to SCH442416, indicating successful encapsulation. Finally, immunostimulatory YCW were integrated onto BSCS@PH to generate BSCS@PHY, designed to couple photothermal activation, A2AR blockade, and innate adjuvanticity within a single construct. TEM revealed BSCS@PHY nanoparticles with an average diameter of 22.7 ± 4.2 nm (Figure S3). EDS detected Se, Bi, Cu together with O and N, consistent with a BSC core plus organic/surface constituents (Figure S4). Both BSC and BSCS@PHY formed visually homogeneous aqueous dispersions (Figure S5). Dynamic light scattering (DLS) in water measured hydrodynamic diameters of ∼51 nm for BSC and ∼93 nm for BSCS@PHY (Figure 2F), the increase being consistent with the added PCMs‐HA coating and YCW layer. The ζ‐potential of BSCS@PHY was ∼‐25 mV (Figure 2G), indicating a negatively charged surface at neutral pH. Powder X‐ray diffraction (XRD) showed that the characteristic reflections of crystalline BSC were retained after assembly into BSCS@PHY (Figure 2H), indicating preservation of the core lattice in the final formulation.

Encouraged by the broad NIR absorbance, we quantified photothermal performance at 808 nm using infrared thermography. Under 1.0 W cm^−2^ irradiation for 10 min, water showed negligible heating, whereas BSC dispersions exhibited concentration‐dependent temperature rises: ΔT ≈ 8.2 °C at 25 µg mL^−1^ increasing to 21.2 °C at 200 µg mL^−1^ (Figure 2I). At fixed concentration, higher laser power densities produced larger ΔT (Figure 2J). BSC also maintained stable heating over four irradiation‐cooling cycles (Figure 2K). BSCS@PHY showed analogous behavior: ΔT scaled with concentration and power, and four on/off cycles did not diminish its output (Figure S6). Together, these data indicate that BSC and BSCS@PHY afford reproducible, laser‐controlled heating suitable for photothermal therapy and thermos‐responsive release.

Reactive oxygen species (ROS) generation was quantified with the 3,3’,5,5’‐tetramethylbenzidine (TMB) assay, in which oxidation yields blue‐green oxidized TMB (ox‐TMB) with an absorbance maximum near 650 nm. At pH 7.4, mixtures of TMB, H_2_O_2_, and BSC‐based formulations produced a clear ∼650 nm band (Figure 2L and Figure S7A). Under mildly acidic conditions (pH 6.5), typical of the TME, the ∼650 nm signal and visible color change were enhanced (Figure 2M and Figure S7B), indicating increased •OH production. Raising the temperature to 37 °C further augmented the ∼650 nm absorbance (Figure S8), consistent with temperature‐accelerated Fenton‐like kinetics. Because elevated GSH can quench ROS [46], we next assessed GSH consumption at pH 6.5. BSC and BSCS@PHY depleted GSH in a dose‐dependent manner (Figure 2N,O). Bismuth selenide (BS) also consumed GSH (Figure S9), consistent with reduction of Se‐Se motifs by GSH; incorporation of copper in BSC increased GSH depletion relative to BS alone [47, 48], consistent with Cu‐mediated redox cycling that both consumes GSH and supports Fenton‐like •OH generation. Together, these data establish that BSCS@PHY supports a customized cascade comprising photothermal heating, GSH depletion, and chemodynamic ROS production.

### 2.2 Evaluation of In‐Vitro Antitumor Efficacy of BSCS@PHY

**FIGURE 3:**
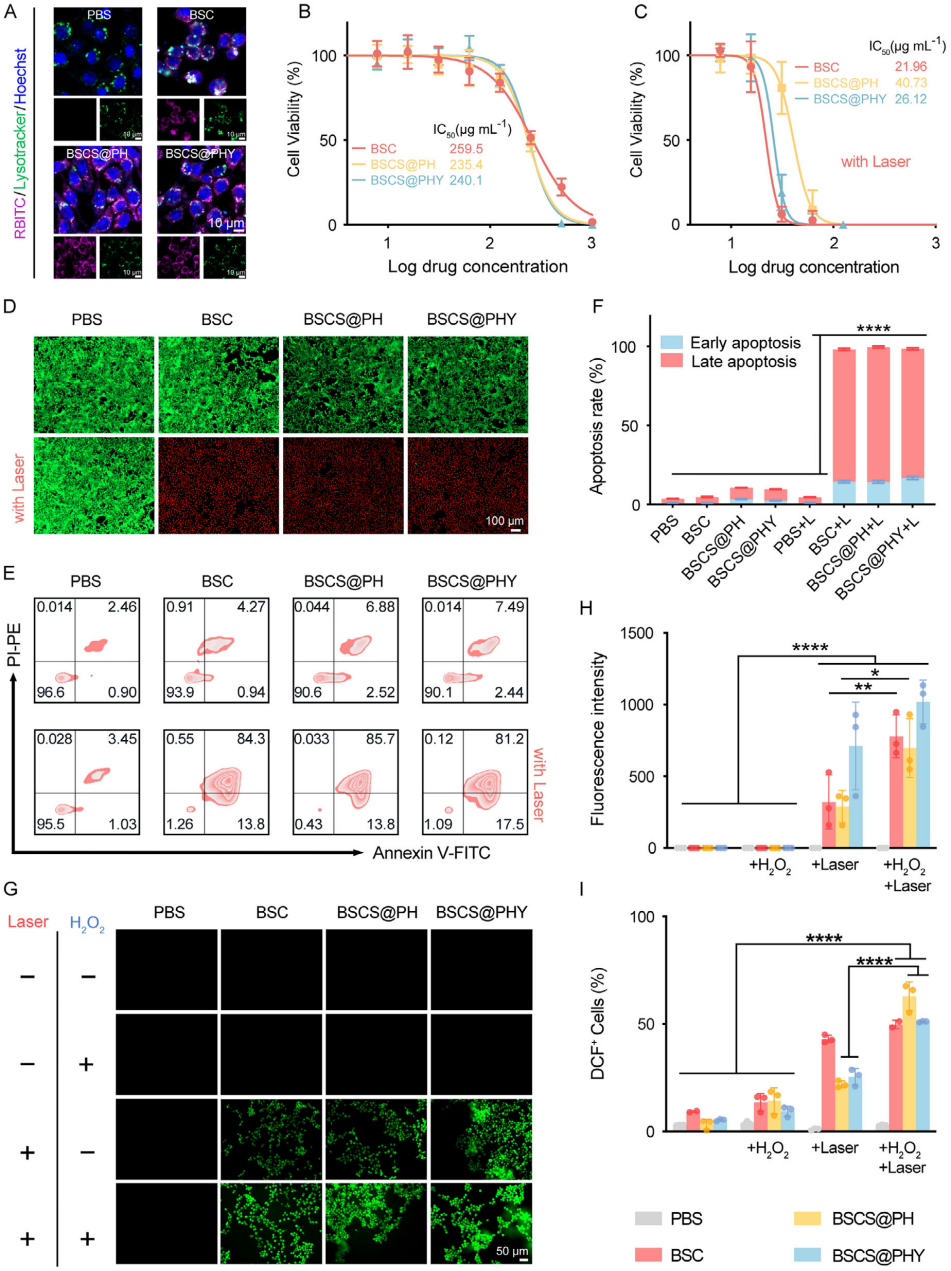
Evaluation of cellular internalization and in‐vitro antitumor activity. (A) Representative fluorescence micrographs of 4T1 cells illustrating uptake of BSC, BSCS@PH, and BSCS@PHY. (B, C) IC_50_ values of the different formulations against 4T1 cells in the absence (B) or presence (C) of NIR laser treatment. (D) Calcein‐AM/propidium iodide (Live/Dead) fluorescence images of 4T1 cells following treatment with the indicated formulations, with and without NIR laser exposure. (E, F) Representative flow cytometric plots and summary quantification of apoptosis in 4T1 cells after different treatments, as determined by Annexin V‐FITC/PI staining. (G, H) Representative fluorescent images and quantitative analysis of intracellular ROS levels in 4T1 cells following different treatments. (I) Flow cytometry analysis of intracellular ROS levels in 4T1 cells after the indicated treatments. Data are presented as means ± SD with n = 3 per group. Statistical significance across groups was assessed using one‐way ANOVA and Tukey's posttest. Significance levels are indicated as follows: ^*^ *P* < 0.05, ^**^ *P* < 0.01, ^***^ *P* < 0.001, ^****^ *P* < 0.0001; n.s. denotes no significance.

To support subsequent efficacy testing, we first verified cellular uptake. After 6 h of incubation with 4T1 cells, RBITC‐labeled BSC, BSCS@PH, and BSCS@PHY showed strong intracellular fluorescence by confocal microscopy, while flow cytometry confirmed efficient cellular uptake of all formulations (Figure 3A and Figure S10). With uptake established, we quantified in vitro cytotoxicity using a cell‐viability assay. In the absence of laser irradiation, all formulations decreased 4T1 cell viability in a concentration‐dependent manner (Figure 3B,C and Figure S11), with half‐maximal inhibitory concentration (IC_50_) values of 259.5 µg mL^−^^1^ for BSC, 235.4 µg mL^−^^1^ for BSCS@PH, and 240.1 µg mL^−^^1^ for BSCS@PHY. Near‐infrared laser exposure markedly increased cytotoxicity for each formulation (Figure 3C and Figure S11); BSC yielded the lowest IC_50_ (21.96 µg mL^−^^1^), consistent with efficient photothermal conversion by the BSC core, and BSCS@PHY (26.12 µg mL^−^^1^) outperformed BSCS@PH (40.73 µg mL^−^^1^). This difference may reflect formulation‐specific responses to heating.

To visualize treatment effects, we performed Calcein‐AM/propidium iodide (PI) live‐dead staining (Figure 3D). Without NIR, PBS‐ and BSC‐treated cells showed predominantly green calcein fluorescence, indicating high viability, whereas BSCS@PH and BSCS@PHY yielded weaker green signals, consistent with the reduced viability in Figure 3B. After NIR exposure (1 W cm^−2^, 10 min), cells treated with BSC, BSCS@PH, or BSCS@PHY exhibited intense PI fluorescence with markedly diminished calcein signal, consistent with extensive loss of viability following laser activation. Flow‐cytometric apoptosis assays corroborated these findings: post‐irradiation, the apoptotic fraction exceeded 98% for each formulation (Figure 3E,F). Together, imaging and cytometry confirm robust laser‐enhanced cytotoxicity of the BSC‐based systems.

To determine whether BSCS@PHY could elevate intracellular oxidative stress, intracellular ROS levels were evaluated using a cell‐permeable fluorogenic probe, followed by fluorescence microscopy and flow cytometry. After 5 h of incubation, cells treated with PBS, BSC, BSCS@PH, or BSCS@PHY showed no appreciable fluorescence above the background level, with or without H_2_O_2_ (Figure 3G,H), indicating limited intracellular ROS accumulation before NIR activation. In contrast, subsequent NIR irradiation (1 W cm^−^^2^, 10 min) markedly increased the fluorescence signal in the nanoparticle‐treated groups, suggesting laser‐triggered ROS generation. Notably, under NIR irradiation, the addition of H_2_O_2_ further increased intracellular ROS levels, particularly in the BSCS@PH and BSCS@PHY groups, supporting H_2_O_2_‐dependent catalytic ROS amplification in the staged nanosystem (Figure 3I). Mechanistically, these results suggest that NIR‐induced thermal activation promotes exposure of the catalytic BSC core, facilitates GSH‐responsive diselenide cleavage, and enables Cu‐mediated Fenton‐like reactions, thereby amplifying intracellular oxidative stress. This ROS elevation may contribute to tumor cell damage and further support the induction of ICD.

Depleting intracellular GSH compromises antioxidant buffering and sensitizes cells to oxidative injury; concurrently, Cu released from BSC can catalyze Fenton‐like reactions in the presence of H_2_O_2_ to generate cytotoxic •OH. Consistent with this mechanism, 4T1 cells exposed to BSC, BSCS@PH, or BSCS@PHY showed significant decreases in both total GSH and reduced GSH (Figure S12), indicating robust GSH consumption [49]. These data support BSC‐based nanoparticles—particularly BSCS@PHY—as candidates for combination regimens that leverage GSH depletion and chemodynamic ROS generation to enhance tumor cell killing.

### 2.3 BSCS@PHY Cascade Therapy Elicits DNA Damage and Immunogenic Cell Death in Vitro

**FIGURE 4:**
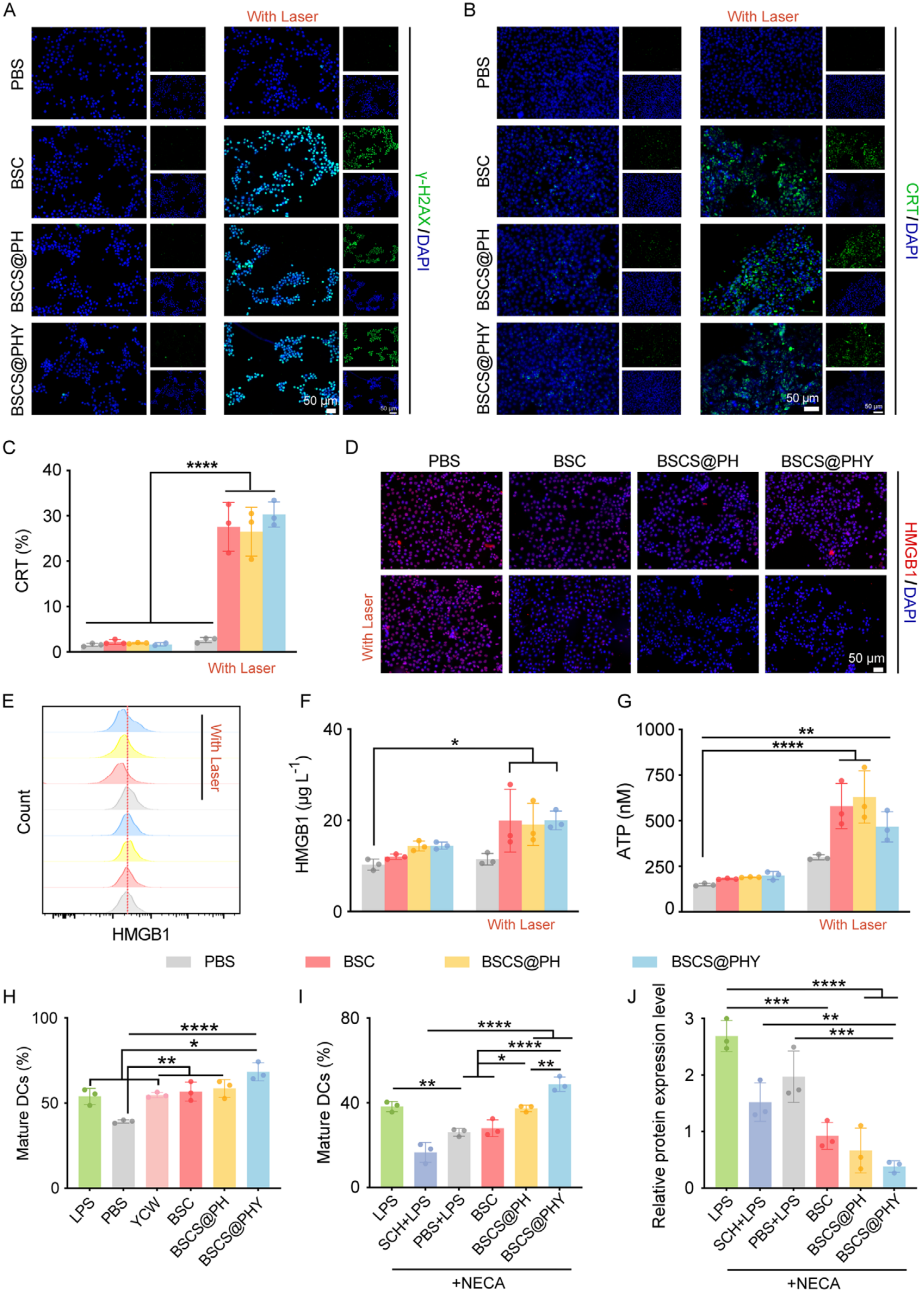
BSCS@PHY‐mediated customized cascade therapy induces DNA damage and ICD in vitro, enhances DC maturation, and suppresses the adenosine‐A2AR pathway in DCs. (A) Broken DNA strands in 4T1 cells stained using the 𝛾‐H2AX assay across different treatment groups. (B) Representative fluorescent microscopy images showing CRT exposure on 4T1 cells. (C) Flow cytometric analysis of CRT exposure on 4T1 cells following different treatments. (D) Representative fluorescent microscopy images depicting HMGB1 release in 4T1 cells after treatments. (E) Representative flow cytometry results showing HMGB1 expression in tumor cells after the indicated treatments. (F) Quantitative analysis of extracellular HMGB1 levels. (G) Quantification of extracellular ATP levels in 4T1 cells after different treatments. (H) Flow cytometry analysis of BMDC maturation after treatment with different formulations. (I) Flow cytometric analysis of BMDC maturation following various treatments. (J) Densitometric quantification of total A2AR protein levels from Western blot analysis normalized to GAPDH. Data are presented as means ± SD, n = 3 per group. Statistical significance was assessed using one‐way ANOVA and Tukey's posttest. Significance levels are indicated as follows: ^*^ *P* < 0.05, ^**^ *P* < 0.01, ^***^ *P* < 0.001, ^****^ *P* < 0.0001; n.s. denotes no significance.

We next assessed DNA double‐strand break (DSB) signaling using 𝛾‐H2AX immunofluorescence [50, 51]. Without NIR irradiation, cells exposed to BSC, BSCS@PH, or BSCS@PHY showed minimal nuclear 𝛾‐H2AX signal (Figure 4A and Figure S13), indicating a low basal DSB burden. Upon NIR exposure (1 W cm^−2^, 10 min), all three formulations induced pronounced nuclear 𝛾‐H2AX foci, consistent with treatment‐evoked DNA‐damage. These findings support that BSCS@PHY, when paired with NIR, elicits a robust DNA‐damage response in tumor cells, strengthening its therapeutic rationale.

Next, we investigated whether BSCS@PHY could stimulate ICD, a process that can enhance antitumor immunity. We assessed three key ICD biomarkers: CRT exposure on the cell surface, high mobility group box 1 (HMGB1) release, and ATP secretion [52, 53]. CRT exposure and HMGB1 release were assessed through fluorescent imaging and flow cytometry (Figure 4B–E and Figure S14). In the absence of NIR laser activation, cells treated with BSC, BSCS@PH, and BSCS@PHY exhibited only faint CRT fluorescence on the cell membrane (Figure 4B and Figure S14A), indicating limited induction of CRT exposure without NIR stimulation. In contrast, following NIR laser irradiation, the same formulations triggered substantial CRT exposure on the cell membrane, as evidenced by strong green fluorescence. These results demonstrate that NIR‐triggered BSCS@PHY therapy significantly enhances CRT exposure. Further validation through flow cytometry confirmed that treatment with BSC, BSCS@PH, and BSCS@PHY in conjunction with NIR laser irradiation significantly increased CRT exposure (Figure 4C and Figure S14B). HMGB1 release was evaluated by immunofluorescence. Without NIR, cells exposed to BSC, BSCS@PH, or BSCS@PHY showed retained intracellular HMGB1 with only modest signal reduction (Figure 4D and Figure S14C). With NIR activation, all three formulations produced marked loss of intracellular HMGB1 staining (diminished red signal), consistent with HMGB1 release/relocation from nuclei to the extracellular space. Flow cytometry concordantly showed a significant decrease in intracellular HMGB1 mean fluorescence in the NIR‐treated groups (Figure 4E and Figure S14D). Quantitative analysis of HMGB1 and ATP levels confirmed that the combination of NIR laser and BSC, BSCS@PH, or BSCS@PHY significantly increased both HMGB1 and ATP secretion compared to the control group (Figure 4F,G). These results collectively demonstrate that BSCS@PHY, when activated by NIR laser, efficiently induces ICD in tumor cells, thereby enhancing its potential for immunotherapeutic applications.

### 2.4 BSCS@PHY is Associated with Relief of A2AR‐Mediated Suppression and Promotes DC Maturation

DCs serve as the principal population of APCs and are central to the initiation of adaptive immune responses. Given their importance in immune activation, we evaluated the maturation of bone marrow‐derived dendritic cells (BMDCs) following exposure to various formulations. Treatments with LPS, YCW, BSC, BSCS@PH, and BSCS@PHY significantly enhanced the expression of co‐stimulatory molecules (CD80 and CD86) compared to PBS (Figure 4H). Notably, BSCS@PHY induced the highest levels of CD80 and CD86, underscoring its robust efficacy in promoting BMDC maturation.

In the TME, adenosine accumulation suppresses immunity via A2AR on immune cells [54, 55]; blocking A2AR reverses this inhibition and enhances antitumor responses [8, 56]. Therefore, we investigated the potential of BSCS@PHY‐mediated cascade therapy to enhance DC maturation by inhibiting the Adenosine‐A2AR pathway. To better simulate the impact of BSCS@PHY on immune cells in cancer therapy, we first collected supernatants from 4T1 cells treated with various formulations combined with laser irradiation. These supernatants were then used to treat immature BMDCs. The experimental conditions included LPS (positive control), supernatants from (PBS+Laser)‐treated cells combined with LPS and NECA (adenosine analog and a potent and non‐selective adenosine receptor agonist [57, 58]) (PBS+LPS+NECA), supernatants from (SCH+Laser)‐treated cells combined with LPS and NECA (SCH+LPS+NECA), supernatants from (BSC+Laser)‐treated cells combined with NECA (BSC+NECA), supernatants from (BSCS@PH+Laser)‐treated cells combined with NECA (BSCS@PH+NECA), and supernatants from (BSCS@PHY+Laser)‐treated cells combined with NECA (BSCS@PHY+NECA) (Figure 4I). Relative to the LPS positive control, the PBS+LPS+NECA group showed a significant reduction in BMDC maturation, indicating that supernatant from PBS plus laser–treated cells, even with LPS, does not overcome NECA‐mediated suppression via adenosine receptors. The SCH+LPS+NECA group likewise did not increase the fraction of mature BMDCs. In contrast, BSCS@PH+NECA and BSCS@PHY+NECA yielded significantly higher fractions of mature BMDCs than BSC+NECA. This pattern is consistent with formulation differences: BSCS@PH contains the A2AR antagonist SCH442416 and PCMs‐HA, which can mitigate adenosine signaling and enable thermally triggered release, whereas BSCS@PHY further includes yeast cell wall that provides innate adjuvanticity. Notably, BSCS@PHY+NECA achieved the highest maturation, consistent with concurrent A2AR antagonism and β‐glucan‐driven stimulatory cues present in the tumor‐conditioned supernatant. To investigate the mechanism, we quantified A2AR protein levels in BMDCs by immunoblotting. The BSCS@PHY+NECA group showed lower total A2AR protein levels, with significant differences observed compared with the LPS, PBS+LPS+NECA, and SCH+LPS+NECA groups (Figure 4J and Figure S15). Although these results do not establish direct causality, they are consistent with attenuation of A2AR‐mediated suppressive signaling during BSCS@PHY‐mediated cascade therapy and are in line with the enhanced DC maturation observed above. Collectively, these findings support the interpretation that BSCS@PHY promotes DC maturation, at least in part, by limiting A2AR‐mediated suppression, which may further strengthen antitumor immunity.

### 2.5 Biodistribution Analysis and Photothermal Imaging of BSCS@PHY

**FIGURE 5:**
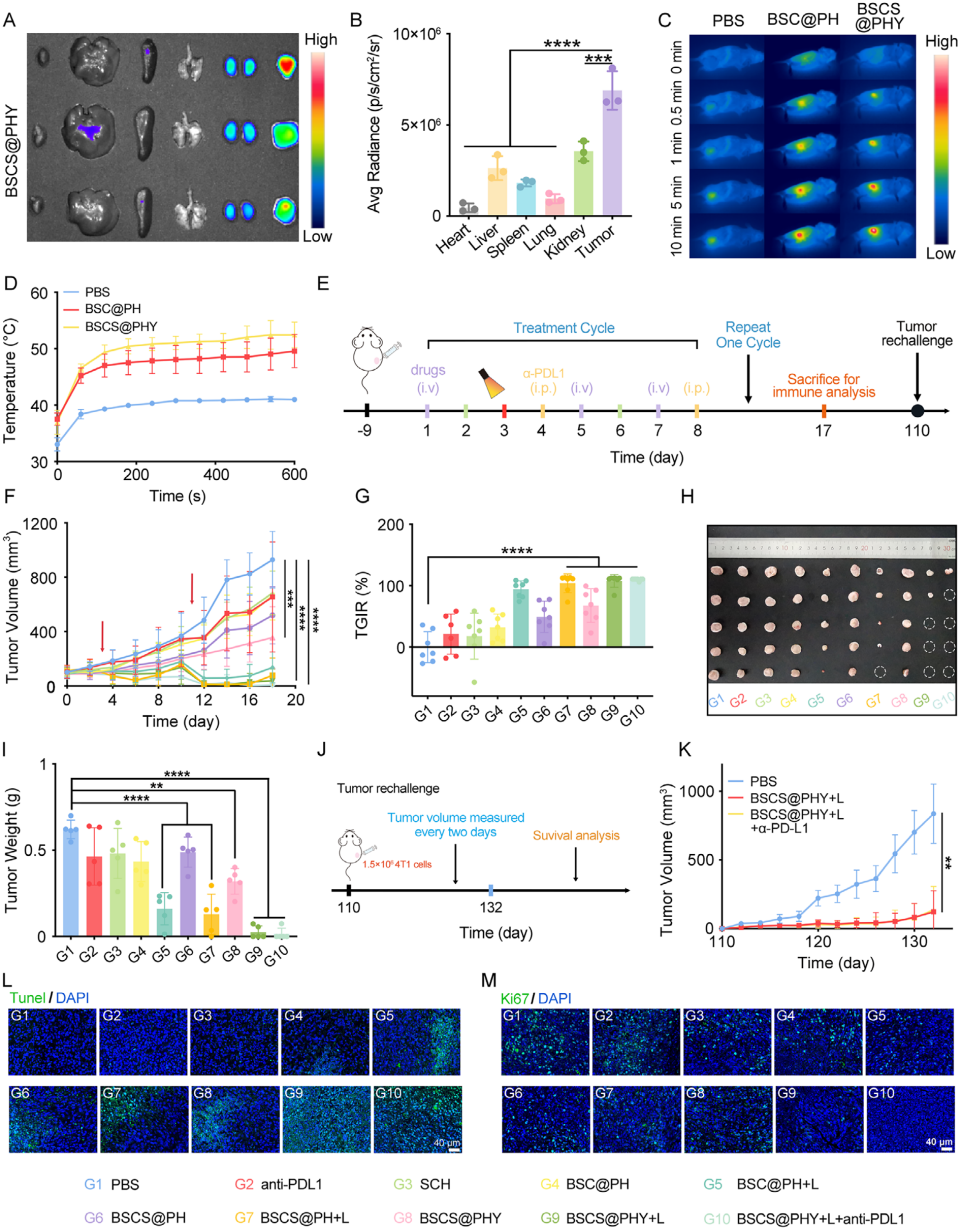
Assessment of antitumor efficacy in vivo. (A) Time‐course fluorescent imaging of tumor‐bearing mice after intravenous administration of ICG‐labeled BSCS@PHY. (B) Quantitation of the accumulation in tumors and major organs (n = 3). (C) Representative photothermal images of mice after intravenous administration of different formulations. (D) Temperature profiles at the tumor site following intravenous administration of various formulations (n = 3). (E) Schematic illustration of the in vivo experimental design. (F) Tumor growth kinetics in mice receiving different treatment regimens (n = 7). The arrow marks the day on which laser irradiation was performed. (G) TGIR in mice receiving the indicated formulations (n = 6–7). (H, I) Photos and weights of excised tumor tissues from mice after different treatments (n = 5). (J) Schematic representation of the tumor rechallenge study design. (K) Tumor growth curves of mice (n = 8). Representative images of tumor tissues from mice after different treatments. (L, M) TUNEL assays and Ki67 staining of tumor tissues. Data are presented as means ± SD. Statistical significance across groups was assessed using one‐way ANOVA followed by Tukey's post‐hoc test. Significance levels are indicated as follows: ^**^ *P* < 0.01, ^***^ *P* < 0.001, ^****^ *P* < 0.0001.

Prior to in vivo antitumor efficacy assessment of BSCS@PHY, we examined its tumor‐targeting behavior and biodistribution in BALB/c mice bearing murine triple‐negative breast cancer (TNBC) tumors. For fluorescence tracking, BSCS@PHY was labeled with indocyanine green (ICG) and injected intravenously into 4T1 tumor‐bearing mice at a dose of 20 mg kg^−1^. 72 h post‐injection, tumors and main organs were collected for ex vivo fluorescence imaging. Strong fluorescence was observed in tumor tissues compared with other organs (Figure 5A,B), indicating a significant accumulation of BSCS@PHY at the tumor site. Fluorescent signals were also detected in the liver and kidneys, suggesting that BSCS@PHY may undergo metabolism through hepatic and renal pathways. Additionally, 36 h after intravenous injection, tumor‐bearing mice were treated with NIR laser irradiation for 10 min (Figure 5C,D). While only minor temperature changes were observed in the PBS group, the temperature at the tumor site in the BSCS@PHY‐treated group increased to approximately 52 °C after NIR laser exposure, confirming the superior photothermal efficacy of BSCS@PHY.

### 2.6 BSCS@PHY Cascade Therapy Suppresses Progression of Triple‐Negative Breast Tumors

Next, we investigated the potential application of BSCS@PHY for tumor therapy in vivo, using the 4T1 TNBC mouse model, which is characterized by rapid tumor progression and high metastatic potential [32, 59]. In this study, we assessed the antitumor efficacy of BSCS@PHY‐mediated customized cascade therapy, both as a standalone treatment and in combination with anti‐PD‐L1, a well‐established immune checkpoint inhibitor. The treatment protocol is outlined in Figure 5E. When tumor reached 100–150 mm^3^, mice were divided into 10 cohorts (n = 7): PBS (G1), anti‐PD‐L1 (G2), SCH442416 (G3, SCH), BSC@PH (G4), BSC@PH+Laser (G5, BSC@PH+L), BSCS@PH (G6), BSCS@PH+Laser (G7, BSCS@PH+L), BSCS@PHY (G8), BSCS@PHY+Laser (G9, BSCS@PHY+L), BSCS@PHY+Laser+anti‐PD‐L1 (G10, BSCS@PHY+L+anti‐PD‐L1). Tumor volume and body weight were monitored every two days during the treatment period. The results demonstrate the efficacy of BSCS@PHY‐mediated combination therapy (Figure 5F–I, Figures S16 and S17). In the group treated with BSC@PH combined with NIR laser (G5), tumor growth was initially suppressed, but the tumors began to regrow over time, suggesting that PTT and enhanced CDT alone were not sufficient for sustained tumor inhibition. In contrast, mice treated with BSCS@PHY (G8) had significantly slower tumor growth, likely due to the gradual degradation of BSCS@PHY within the TME, which enhanced CDT and facilitated the release of SCH442416 and yeast cell wall components. This combination resulted in a tumor growth inhibition rate (TGIR) of ∼68% (Figure 5G). Tumor growth was further suppressed in the BSCS@PHY+Laser group (G9), achieving a TGIR of over 95%. Remarkably, the combination of BSCS@PHY with NIR laser and anti‐PD‐L1 therapy (G10) achieved a TGIR exceeding 98%, with complete tumor elimination observed in 86% of the treated mice. Additionally, measurements of tumor size and weight were consistent with these results (Figure 5H,I), highlighting the antitumor efficacy of BSCS@PHY‐based cascade therapy. Aligned with this, the survival rates significantly improved in the BSCS@PHY+Laser (G9) and BSCS@PHY+Laser+anti‐PD‐L1 (G10) groups, reflecting the potent antitumor effects of the combination therapy.

In a subsequent rechallenge experiment, mice in the BSCS@PHY+Laser and BSCS@PHY+Laser+anti‐PD‐L1 groups that survived for 110 days were rechallenged with tumor cells (Figure 5J,K and Figure S18). Tumor growth in these rechallenged mice was significantly slower than in the control group. Notably, 37.5% of the mice in the BSCS@PHY+Laser+anti‐PD‐L1 group exhibited complete suppression of tumor regrowth for at least 170 days (Figure S18), suggesting the development of long‐lasting immunity. These results highlight the durability of the immune response and the potential of BSCS@PHY‐based cascade therapy, particularly when combined with immune checkpoint blockade (ICB), as a promising treatment for patients with otherwise inaccessible tumors.

To further confirm the antitumor efficacy, we conducted hematoxylin and eosin (H&E) staining and terminal deoxynucleotidyl transferase dUTP nick end labeling (TUNEL) assays on the excised tumors from mice treated with various therapies. Both BSC@PH and BSCS@PH, when combined with NIR laser treatment, induced significant necrosis within the tumors (Figure 5L and Figure S19A). Notably, BSCS@PHY‐mediated cascade therapy, particularly in combination with anti‐PD‐L1, exhibited even more potent antitumor effects compared with other groups. Furthermore, Ki67 staining, a marker for cell proliferation, revealed negligible fluorescence in tumors from the BSCS@PHY+Laser+anti‐PD‐L1 group (Figure 5M), underscoring a substantial inhibition of tumor cell proliferation. Throughout the therapy period, body weights did not exhibit appreciable variation (Figure S19B), indicating the absence of major systemic toxicity. Additionally, histological examination of major organs and serum biochemical profiling revealed no detectable organ damage or disturbances in hepatic and renal function (Figure S19A,C), further confirming the safety of BSCS@PHY‐based therapy. These findings collectively demonstrate the efficacy, safety, and promising therapeutic potential of BSCS@PHY‐mediated customized cascade therapy for treating TNBC.

### 2.7 BSCS@PHY Cascade Therapy Reprograms the Immune Microenvironment of Triple‐Negative Breast Tumors

To probe mechanisms underlying the activity of BSCS@PHY with laser and anti‐PD‐L1, we analyzed tumor‐draining lymph nodes (TDLNs), tumors, and spleens from treated mice. Given that induction of ICD can remodel immunologically “cold” tumors into inflamed “hot” lesions, we assessed CRT exposure by immunofluorescence. Tumors from BSCS@PHY+Laser and BSCS@PHY+Laser+anti‐PD‐L1 showed strong green fluorescence consistent with CRT translocation (Figure S20A), indicating effective ICD induction in both regimens. ICD releases ATP, which in the TME is rapidly hydrolyzed by CD39/CD73 to adenosine that suppresses immunity through A2AR. To mitigate this brake, our formulations incorporate the A2AR antagonist SCH442416. We therefore examined A2AR in TDLNs by immunofluorescence. Compared with PBS (G1), the groups that contained SCH442416, namely BSCS@PH (G6), BSCS@PH+Laser (G7), BSCS@PHY (G8), BSCS@PHY+Laser (G9), and BSCS@PHY+Laser+anti‐PD‐L1 (G10), displayed lower A2AR immunofluorescence (Figure S20B), with the largest reductions in G9 and G10. Taken together, CRT translocation in tumors indicates enhanced ICD, and reduced A2AR immunofluorescence in TDLNs is consistent with weakened adenosine–A2AR signaling. While these findings do not establish causality, they provide a mechanistic rationale for enhanced antitumor immunity with laser‐triggered BSCS@PHY ± anti‐PD‐L1.

**FIGURE 6:**
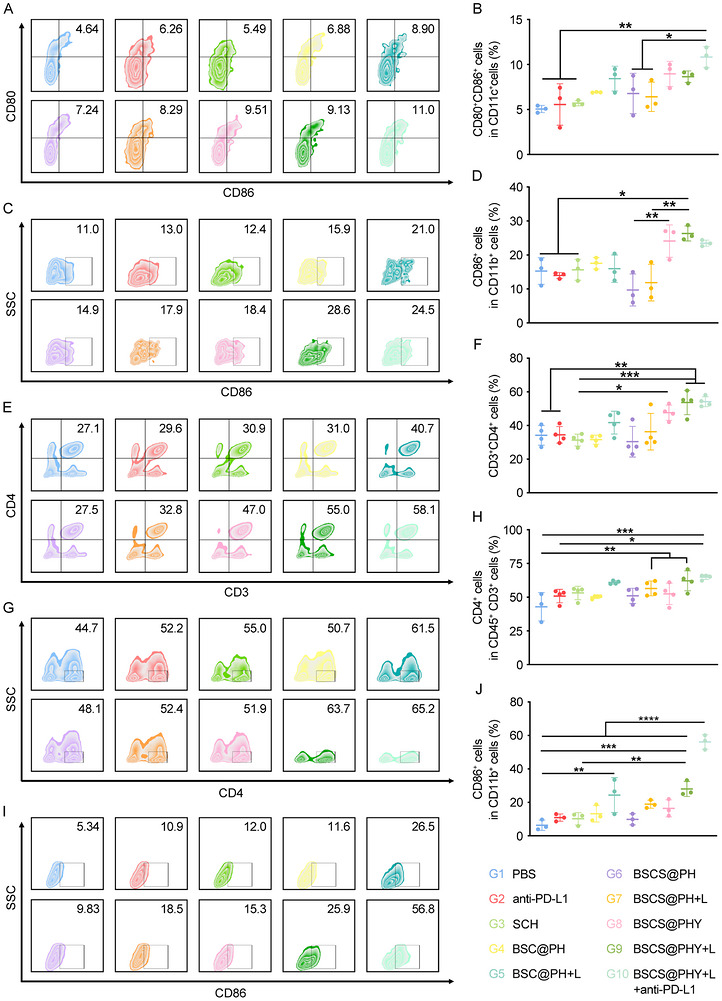
Evaluation of antitumor immunity in vivo. (A‐I) Representative flow cytometric plots and corresponding quantitative analysis showing the percentages of mature DCs in TDLNs (A and B), M1 macrophages in TDLNs (C and D), CD4^+^ T cells in TDLNs (E and F), CD4^+^ T cells in spleens (G and H), and M1 macrophages in tumors (I and J). Data are presented as mean ± SD, n = 3–4. For multi‐group comparisons, one‐way ANOVA followed by Tukey's post hoc test was used. Significance is denoted as ^*^ *P* < 0.05, ^**^ *P* < 0.01, ^***^ *P* < 0.001, ^****^ *P* < 0.0001.

To assess the immune response, we profiled immune cell populations in TDLNs, spleens, and tumors after different treatments using flow cytometry. DCs bridge innate danger sensing with adaptive T‐cell priming [60, 61], and their maturation is essential for effective antitumor immunity, making them key targets for cancer immunotherapy. In TDLNs, BSCS@PHY+Laser+anti‐PD‐L1 increased the fraction of mature DCs (CD80^+^CD86^+^) 2.14‐fold versus PBS (G1), indicating enhanced DC maturation (Figure 6A,B). Macrophage polarization shapes therapy outcomes: M1 macrophages are immunostimulatory and antitumor, whereas M2 macrophages are immunosuppressive and pro‐tumorigenic [15, 62]. In TDLNs, BSCS@PHY+Laser significantly increased the proportion of M1‐like macrophages (CD11b^+^CD86^+^) relative to PBS (G1) (Figure 6C,D). Moreover, BSCS@PHY exceeded BSCS@PH, a pattern consistent with yeast cell‐wall components enhancing myeloid activation and mitigating immunosuppression in the TME. CD4^+^ T cells coordinate antitumor immunity. In TDLNs and spleens, BSCS@PHY+Laser and BSCS@PHY+Laser+anti‐PD‐L1 showed significantly higher CD4^+^ T cell frequencies than PBS (Figure 6E‐H), indicating that BSCS@PHY+Laser, particularly in combination with anti‐PD‐L1, expands helper T cells in secondary lymphoid organs. Additionally, in tumor tissues, the fraction of M1 macrophages was markedly higher in the BSCS@PHY+Laser+anti‐PD‐L1 group compared to other treatment groups (Figure 6I,J). Additionally, immunofluorescence staining showed increased CD8^+^ T cell infiltration in tumors from the BSCS@PHY+Laser (G9) and BSCS@PHY+Laser+anti‐PD‐L1 (G10) groups (Figure S20C). CD4^+^ T cell expression in TDLNs was also notably higher in these groups (Figure S20D). Collectively, these findings indicate that BSCS@PHY combined with laser irradiation and anti‐PD‐L1 therapy reprograms the immune microenvironment, effectively activating a robust antitumor immunity.

**FIGURE 7:**
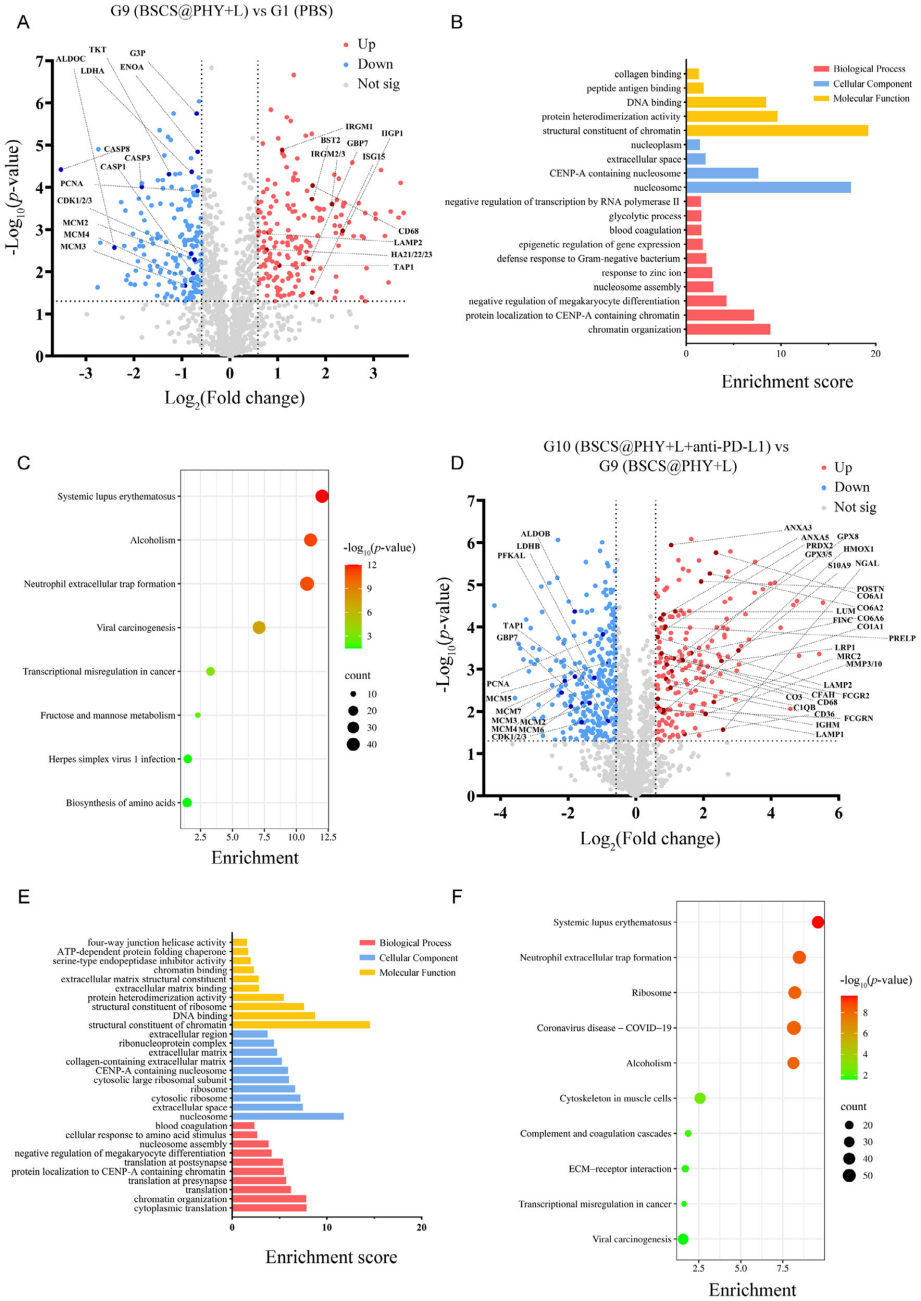
Label‐free quantitative proteomics of whole tumor tissues. (A‐C) G9 (BSCS@PHY+Laser) vs PBS: (A) volcano plot of protein‐level changes; (B) GO enrichment across BP, CC, and MF; (C) KEGG pathway enrichment. (D‐F) G10 (BSCS@PHY+Laser+anti‐PD‐L1) vs G9: (D) volcano plot; (E) GO enrichment (BP/CC/MF); (F) KEGG pathway enrichment. Proteins with a fold change ≥ 1.5 and p ≤ 0.05 were designated as DEPs. For GO bar plots (B, E), the x‐axis shows the enrichment score, and terms are ordered by significance (−log_10_(*p*‐value)). For KEGG bubble plots (C, F), the x‐axis shows the enrichment score, bubble size encodes the number of mapped DEPs, and bubble color encodes −log_10_(*p*‐value). Note that GO/KEGG categories are direction‐agnostic; up/down regulation was inferred from the volcano plots.

We next performed label‐free quantitative proteomic profiling of whole tumor tissues. Compared with PBS, G9 (BSCS@PHY+Laser) induced broad proteomic remodeling, with differentially expressed proteins (DEPs) distributed across both up‐ and down‐regulated categories (Figure 7A). The volcano plot showed coordinated down‐regulation of proteins associated with DNA replication and glycolytic metabolism (PCNA, MCM2/3/4, CDK1/2/3, ALDOC, LDHA, TKT, ENOA, G3P) [63, 64, 65, 66, 67, 68, 69, 70], together with marked up‐regulation of proteins related to interferon responses, antigen processing, and phagocytic/lysosomal activity (IRGM1/2/3, BST2, GBP7, ISG15, IIGP1, TAP1, HA1/2/3, CD68, LAMP2) [71, 72, 73, 74, 75, 76, 77, 78]. Gene Ontology (GO) enrichment highlighted two principal axes (Figure 7B): immune/extracellular processes and chromatin/nucleosome and metabolic programs. Kyoto Encyclopedia of Genes and Genomes (KEGG) pathway enrichment was consistent with these patterns (Figure 7C), with chromatin‐associated, innate immune‐related, and metabolic pathways reflecting these coordinated protein changes. Taken together, BSCS@PHY+Laser generated a proteomic signature consistent with laser‐triggered redox stress and ICD‐associated immune remodeling. Antigen‐processing, phagocytic/lysosomal, and extracellular matrix‐associated features were enhanced, whereas proliferation‐associated chromatin/replication and glycolytic programs were reduced, in line with the immune activation phenotypes observed in TDLNs and spleen.

Compared with G9, G10 (BSCS@PHY+Laser+anti‐PD‐L1) induced a broader proteomic shift with coordinated pathway‐level changes (Figure 7D). Volcano‐plot mapping revealed enhanced up‐regulation of proteins associated with complement activation, humoral immunity, opsonization/phagocytosis, and lysosomal activity, including C1QB, CO3, FCGR2/FCGRN, CFAH, IGHM, CD68, CD36, and LAMP1/2 [77, 78, 79, 80, 81, 82, 83], together with additional phagocytic and clearance‐related mediators such as LRP1 and MRC2 [84, 85]. ECM and adhesion‐related proteins, including CO1A1, CO6A1/6A2/6A6, FINC, POSTN, LUM, PRELP, and MMP3/10 [86, 87, 88, 89], were also further increased. Inflammation‐ and oxidative stress‐associated proteins, including NGAL, S100A9, ANXA3/5, PRDX2, GPX3/5/8, and HMOX1 [90, 91, 92, 93, 94, 95, 96], were likewise up‐regulated. Notably, several IFN‐inducible proteins involved in antigen processing, such as GBP7 and TAP1, which were up‐regulated in G9, were modestly reduced in G10. This pattern may reflect a transition from an acute ICD‐associated IFN response toward an immune state more prominently characterized by complement/opsonic‐phagocytic activity, lysosomal function, and ECM remodeling under PD‐L1 blockade. In parallel, proliferation‐, replication‐, and metabolism‐associated proteins, including MCM2–7, CDK1/2/3, PCNA, and glycolytic enzymes such as ALDOB, LDHB, and PFKAL [63, 64, 65, 97, 98, 99], were further reduced. GO enrichment analysis (Figure 7E) revealed two functional axes: an extracellular/hemostatic axis marked by ECM structural components, collagen‐binding terms, and lysosomal/immune‐handling terms; and a chromatin/translation axis characterized by nucleosome, ribosome, cytosolic translation, and chromatin‐organization modules. KEGG analysis (Figure 7F) supported these trends, with enrichment of pathways related to systemic lupus erythematosus and neutrophil extracellular trap formation, reflecting histone/chromatin‐associated modules, as well as ribosome pathways, ECM‐receptor interaction, and complement/coagulation cascades. Together, the G10 versus G9 comparison indicates three coordinated reprogramming features: strengthened complement/opsonic‐phagocytic and lysosomal activity, expanded ECM/adhesion‐associated remodeling, and sustained reduction of proliferation‐associated chromatin, replication, and biosynthetic programs. These changes are consistent with an additional contribution of PD‐L1 blockade to the immune remodeling initiated by G9.

### 2.8 BSCS@PHY Cascade Therapy Limits Distant Tumor Outgrowth via Systemic Antitumor Immunity

**FIGURE 8:**
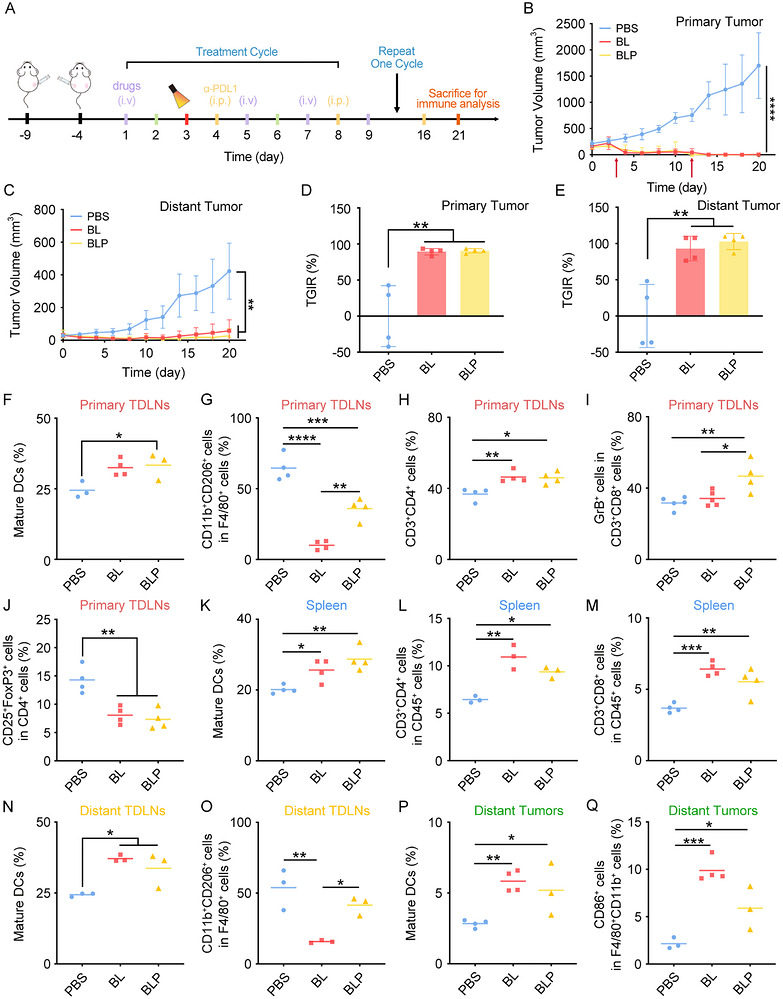
Evaluation of effectiveness in suppressing distant tumor progression. (A) Schematic diagram of treatment protocol in a bilateral 4T1 tumor model. (B) Average growth curves of primary tumor volumes. (C) Average growth curves of distant tumor volumes. (D, E) TGIR for primary tumors (D) and distant tumors (E) in different treatment groups (n = 4). (F‐J) The proportion of mature DCs (F), expression of M2 macrophages (G), percentages of CD4^+^ T cells (H), proportion of CD8^+^GrB^+^ T cells (I), and percentages of Tregs (J) in the primary TDLNs from mice receiving different formulations. (K‐M) The proportion of mature DCs (K), percentages of CD4^+^ T cells (L), and percentages of CD8^+^ T cells (M) in the spleens of mice subjected to various treatments. (N, O) The proportion of mature DCs (N), and M2 macrophage levels (O) in the distant TDLNs of mice with different treatments. (P, Q) The proportion of mature DCs (P) and levels of M1 macrophages (Q) in distant tumors of mice after various treatments. Data are presented as mean ± SD, n = 3–5. For multi‐group comparisons, one‐way ANOVA followed by Tukey's post hoc test was used. Significance is denoted as ^*^ *P* < 0.05, ^**^ *P* < 0.01, ^***^ *P* < 0.001, ^****^ *P* < 0.0001.

Tumor metastasis is a leading cause of treatment failure. Building on the robust control of primary tumors and evidence of treatment‐induced antitumor immunity, we next evaluated whether BSCS@PHY+Laser combined with anti‐PD‐L1 can suppress the growth of distant tumors. To assess abscopal efficacy, we employed a bilateral 4T1 model in which only the primary tumor received NIR irradiation. When primary tumors reached ∼100‐150 mm^3^, mice were randomized (n = 4 per group) to PBS, BSCS@PHY+Laser (BL), or BSCS@PHY+Laser+anti‐PD‐L1 (BLP). The protocol (Figure 8A) applied NIR photothermal therapy only to the primary tumor. As expected, PBS controls showed rapid progression at both sites (Figure 8B,C and Figure S21A,B). In contrast, BL and BLP significantly reduced the growth of both primary and distant tumors, with endpoint volumes markedly lower than PBS (Figure S21C,D). Versus PBS, BL achieved TGIR of approximately 89% at the primary site and ∼93% at the distant site (Figure 8D,E). BLP yielded the greatest suppression, with TGIR ∼ 91% (primary) and ∼99% (distant). These results indicate that local BSCS@PHY‐mediated PTT elicits a systemic antitumor effect and that adding PD‐L1 blockade further enhances control of the unirradiated lesion.

To probe mechanisms underlying distant‐tumor control, we profiled immune cells in TDLNs, spleens, and tumors. In TDLNs draining the primary tumors (referred to as Primary TDLNs), BLP increased mature DCs (CD80^+^CD86^+^) to ∼1.36× that of PBS (Figure 8F and Figure S22A), indicating enhanced DC maturation that supports antitumor priming. The proportion of M2‐like macrophages (F4/80^+^CD11b^+^CD206^+^) was significantly reduced in BL and BLP versus PBS (Figure 8G and Figure S22B), consistent with mitigation of myeloid‐driven immunosuppression. CD4^+^ T cells (CD3^+^CD4^+^) were ∼1.26× (BL) and ∼1.25× (BLP) of PBS (Figure 8H and Figure S22C). Granzyme B‐positive CD8^+^ T cells (GrB^+^CD8^+^ T cells)—a cytotoxicity readout—were significantly higher in BLP than PBS (Figure 8I and Figure S22D). Tregs (CD4^+^Foxp3^+^) decreased to ∼0.57× (BL) and ∼0.51× (BLP) of PBS (Figure 8J and Figure S21E). Taken together, the increased CD4^+^ T‐cell population and GrB^+^CD8^+^ T cells, together with reduced Tregs, suggest a lymph‐node immune milieu more favorable for antitumor responses. In the spleen, both BL and BLP groups showed significantly higher frequencies of mature DCs (CD80^+^CD86^+^) as well as CD4^+^ and CD8^+^ T cells compared with PBS (Figure 8K–M and Figure S23). The BL group also exhibited a significantly increased proportion of M1‐like macrophages relative to PBS (Figure S24). Taken together, these changes are consistent with systemic immune activation. In TDLNs draining distant tumors (referred to as Distant TDLNs), the frequency of mature DCs (CD80^+^CD86^+^) was ∼1.52× (BL) and ∼1.38× (BLP) that of PBS (Figure 8N and Figure S25A), indicating enhanced DC maturation. The proportion of M2‐like macrophages (F4/80^+^CD11b^+^CD206^+^) was significantly lower with BL than with PBS (Figure 8O and Figure S25B). In these nodes, Tregs (CD4^+^Foxp3^+^) decreased to ∼0.68× of PBS with BL (Figure S26), consistent with a reduced immunosuppressive tone in the distant TME. Central memory T (T_CM_) cells contribute to long‐term immune surveillance and are associated with durable antitumor immune protection. T_CM_ cells increased to ∼1.51× of PBS after BLP (Figure S27). Taken together, increases in antigen‐presenting and memory compartments, alongside fewer suppressive cells, support an abscopal, pro‐immunity phenotype. BL and BLP also increased the fraction of mature DCs (CD80^+^CD86^+^) and M1‐like macrophages within distant tumors (Figure 8P,Q and Figure S28), consistent with broader immune activation beyond the irradiated site. Collectively, these data indicate that BL and BLP induce systemic immune activation and are associated with reduced outgrowth of distant tumors in the 4T1 model. These findings suggest that coupling ICD induction with A2AR blockade and yeast‐cell‐wall–driven APC stimulation, with or without PD‐L1 inhibition, may limit metastatic progression and support more durable tumor control.

### 2.9 BSCS@PHY–Enabled Cascade Therapy Elicits Systemic Immunity and Constrains Pulmonary Metastases

**FIGURE 9:**
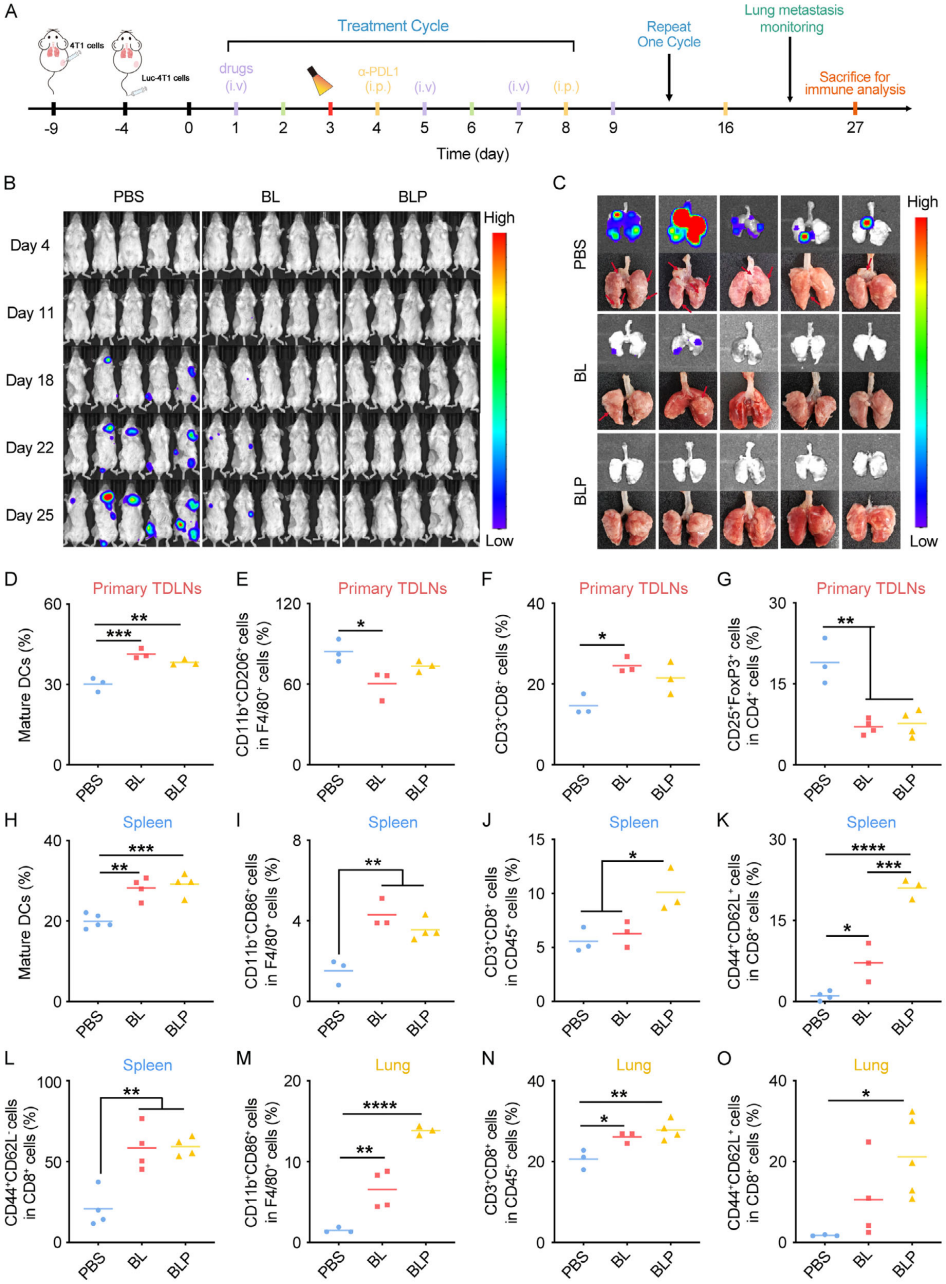
Evaluation of lung metastasis inhibition efficacy. (A) Schematic diagram of the treatment protocol. (B) In vivo bioluminescence images of 4T1 tumor‐bearing mice across treatment groups. (C) Bioluminescence imaging and digital photographs of lungs from mice after different treatments. (D‐G) Levels of mature DCs (D), M2 macrophages (E), CD8^+^ T cells (F), and Tregs (G) in TDLNs from mice receiving different treatments. (H‐L) Percentages of mature DCs (H), M1 macrophages (I), CD8^+^ T cells (J), T_CM_ cells (K), and T_EM_ cells (L) in the spleens of treated mice. (M‐O) Proportions of M1 macrophages (M), CD8^+^ T cells (N), and T_CM_ cells (O) in the lungs of mice given different treatments. Data are presented as mean ± SD, n = 3–5. For multi‐group comparisons, one‐way ANOVA followed by Tukey's post hoc test was used. Significance is denoted as ^*^ *P* < 0.05, ^**^ *P* < 0.01, ^***^ *P* < 0.001, ^****^ *P* < 0.0001.

Lung metastasis is a frequent and lethal complication of breast cancer. To evaluate the anti‐metastatic efficacy of BLP (BSCS@PHY+Laser+anti‐PD‐L1) in the lung, we constructed a luciferase‐based pulmonary metastasis model by intravenously injecting Luc‐4T1 cells into BALB/c mice bearing 4T1 tumors (Figure 9A). PBS controls exhibited strong bioluminescent signals consistent with extensive lung colonization (Figure 9B,C and Figure S29). In contrast, BL (BSCS@PHY+Laser) produced markedly weaker signals, indicating reduced metastatic burden. BLP yielded the lowest signals, consistent with the most pronounced suppression of lung metastasis.

To explore mechanisms underlying anti‐metastatic activity, we profiled immune‐cell subsets in TDLNs, spleens, and lungs by flow cytometry across the treatment groups. In TDLNs, the frequency of mature DCs (CD80^+^CD86^+^) was significantly higher in BL and BLP than in PBS (Figure 9D and Figure S30A): ∼1.38× (BL) and ∼1.27× (BLP) of PBS. These data are consistent with enhanced DC activation/accumulation in TDLNs, a prerequisite for effective priming. M2‐like macrophages (F4/80^+^CD11b^+^CD206^+^) were significantly reduced with BL versus PBS (Figure 9E and Figure S30B), indicating a less immunosuppressive nodal milieu. In the same nodes, CD8^+^ and CD4^+^ T cells were significantly higher in BL than PBS; BLP also increased CD4^+^ T cells (Figure 9F and Figure S30C,D). Tregs (CD4^+^Foxp3^+^) decreased to ∼0.37× (BL) and ∼0.41× (BLP) of PBS (Figure 9G and Figure S30E), further aligning with reduced immunosuppressive tone. In the spleen, the frequency of mature DCs (CD80^+^CD86^+^) was significantly higher in BL and BLP than in PBS (∼1.42× and ∼1.46× of PBS, respectively; Figure 9H and Figure S31A), consistent with systemic DC activation. M1‐like macrophages (e.g., CD11b^+^CD86^+^) were likewise increased with BL and BLP versus PBS (Figure 9I and Figure S31B). In BLP, the spleen showed significant elevations in CD8^+^ T cells, T_CM_, effector memory T cells (T_EM_ cells; cells that provide rapid recall effector responses upon antigen re‐encounter and are associated with immediate antitumor immune protection), and CD4^+^ T cells (Figure 9J–L, Figures S31C–E and S32), consistent with expansion of effector and memory T‐cell compartments. In the lungs, BL and BLP showed significantly higher frequencies of M1‐like macrophages and CD8^+^ T cells than PBS (Figure 9M,N and Figure S33A,B). T_CM_ cells were also significantly increased in BLP versus PBS (Figure 9O and Figure S33C). These correlative immune shifts, particularly the enrichment of M1‐like macrophages and effector T cells in the lung, are consistent with BL‐ and BLP‐induced systemic antitumor immunity, which may contribute to control of the primary lesion and restraint of lung metastases.

## 3 Conclusion

This work introduces a material‐encoded scheduling strategy that synchronizes ICD with receptor‐level relief of adenosine–A2AR suppression in the same tumor niches where priming begins. BSCS@PHY integrates a BSC core (photothermal heating, GSH depletion, Fenton‐like •OH), PCMs‐HA, the A2AR antagonist SCH442416, and YCW to achieve three often‐missing elements in ICD‐only or asynchronous regimens: co‐localization, temporal coordination, and image‐gated control. Across in‐vitro and in‐vivo assays, this triad deepened ICD hallmarks, dampened adenosine‐axis readouts in lymphoid tissues, and expanded antigen‐presenting and effector compartments, translating into control of primary tumors, abscopal restraint of untreated lesions, and reduced pulmonary metastases in 4T1. Mechanistically, thermography‐guided NIR irradiation melts the PCM shell, exposes the catalytic BSC surface, and triggers GSH‐responsive diselenide cleavage with Cu‐mediated •OH generation, thereby amplifying ICD (CRT exposure, HMGB1 release). In the same window, SCH442416 blunts A2AR signaling while YCW provides pathogen‐recognition cues that license DCs. Consistent with this sequence, whole‐tumor proteomics showed that G9 (BSCS@PHY+Laser) induced a remodeling pattern compatible with laser‐triggered redox stress and ICD‐associated immune activation, with enhanced antigen‐processing/phagocytic features and reduced proliferation‐ and glycolysis‐associated programs. The addition of PD‐L1 blockade further strengthened complement/opsonic‐phagocytic and lysosomal pathways, expanded ECM/adhesion remodeling, and maintained suppression of proliferative and biosynthetic programs. Concordantly, immune phenotypes across the TDLNs, spleen, and lung indicated enhanced systemic antitumor immunity. Module comparisons showed nonredundant contributions: removing the A2AR antagonist and/or YCW attenuated DC maturation and immune remodeling versus BSCS@PHY. Photothermal activation functions not only as cytotoxic input but as a reproducible on‐switch that enforces the intended sequence—ICD amplification, antagonist delivery, and innate licensing—thereby overcoming timing mismatches that can blunt ICD‐only approaches [11, 54]. Relative to systemic, unsynchronized A2AR dosing, this framework confines a fast‐acting antagonist to APC‐rich niches and times availability to the brief ATP→adenosine surge [54]; relative to CD39/CD73 inhibition, it targets the convergent receptor node without broadly perturbing ectonucleotidase homeostasis. In the aggressive 4T1 model, image‐gated local activation was associated with immune remodeling in the spleen and in lymph nodes draining both primary and distant tumors, together with a reduced pulmonary metastatic burden. Although these findings are correlative, their reproducible concordance across tissues and endpoints supports a working model whereby temporally synchronized local cues can seed systemic antitumor control. Future work should strengthen causal links (for example, through time‐resolved adenosine measurements, A2AR occupancy assays, and cAMP/PKA signaling readouts), calibrate in vivo release profiles against ICD kinetics, and broaden toxicology, clearance, and model scope. Given its modularity, including tunable PCM thresholds, adaptable catalytic cores, and interchangeable checkpoint or adjuvant modules, BSCS@PHY offers a practical blueprint for converting transient ICD signals into durable systemic immunity and for rationally combining with PD‐(L)1 blockade.

## 4 Methods

### 4.1 Preparation of BSC

5 mL of deionized (DI) water was introduced into a beaker containing 160 mg of selenium powder and 250 mg of polyvinylpyrrolidone (PVP, molecular weight 10 000), then stirred until the selenium powder was evenly dispersed, forming a selenium powder suspension. In a separate beaker, 375 mg of sodium hydroxide was dissolved in 5 mL of DI water, and 75 mg of sodium borohydride (NaBH_4_) was added with stirring to form an alkaline NaBH_4_ solution. This NaBH_4_ solution was then added dropwise to the selenium powder suspension while keeping the mixture on ice. The mixture was sealed and heated at 70 °C for 20 min to obtain a clear, transparent solution. Next, the clear solution was added dropwise to a microwave reaction vial containing 2 mL of water and 90 mg of Bi(NO_3_)_3_, with continuous stirring. Subsequently, 2 mL of a 45 mg mL^−1^ CuCl_2_ solution was introduced, and the mixture was stirred continuously. The resulting mixture was placed in a microwave reactor and heated at 100 °C for 5 min, followed by 180 °C for 10 min, resulting in the formation of a black product. Afterward, the reaction mixture was centrifuged at 14 000 rpm for 10 min and washed three times with DI water to obtain the crude product. The crude product was resuspended in DI water and allowed to stand at room temperature overnight. The supernatant was collected, and the large gray precipitate was discarded, yielding the BSC nanoparticles. For the synthesis of bismuth diselenide (BS) nanoparticles, the same procedure was followed, but without the addition of CuCl_2_.

### 4.2 Synthesis of BSCS@PHY

10 mL of Luria‐Bertani (LB) medium was added to a 50 mL centrifuge tube, and an appropriate amount of yeast suspension was introduced. The tube was incubated in a shaking incubator at 200 rpm and 37 °C for 48 h. Once the optical density (OD) value of the yeast reached 0.8‐1.2, the yeast was collected for further processing. The collected yeast was incubated with 1 M NaOH at 80 °C for 1 h, then naturally cooled to room temperature. Afterward, the lysed yeast was centrifuged at 2000 g for 10 min to obtain the precipitate containing yeast cell walls. The precipitate was washed successively with DI water, isopropanol, and acetone, followed by two additional washes with DI water. The obtained precipitate was then subjected to ultrasonication (setup: 100% power, 10 s pulse, 5 s stop, for a total of 4 cycles). Afterward, the mixture was centrifuged at 2400 g for 10 min to obtain the yeast cell walls, which were stored at ‐20 °C for further use.

Next, 20 mg of BSC nanoparticles were introduced into an aqueous solution of DSPE‐PEG‐NHS (20 mg), and the suspension was stirred for 10 min. Then, 100 µL of a 50 mg mL^−1^ SCH442416 solution was added and stirred at room temperature for 2 h to obtain a BSCS solution.

Hyaluronic acid (HA) (40 mg) and EDC (80 mg) were dissolved separately in DI water. The dissolved HA solution was mixed with the EDC solution and stirred for 15 min to activate HA. Afterward, 40 mg of DSPE‐PEG‐NH_2_ was added to the activated HA solution, and the mixture was stirred for 30 min to obtain the DSPE‐PEG‐NH_2_‐HA solution. In a separate step, 40 mg of phase‐change material (PCM) 1‐tetradecanol was dissolved in absolute alcohol, and this solution was then introduced into the DSPE‐PEG‐NH_2_‐HA solution. The mixture was sealed and incubated in a water bath at 40 °C for 30 min to obtain the PCMs‐HA solution. The BSCS solution was then added dropwise to the PCMs‐HA solution and incubated in a water bath at 40 °C for 2 h to yield the BSCS@PH. The product was purified by centrifugation at 9000 rpm for 10 min, followed by three washes with water to obtain the purified BSCS@PH (SCH442416 loading content: ∼25%). For BSC@PH, an identical protocol was used, excluding the addition of SCH442416. Finally, yeast cell walls and BSCS@PH were mixed at a mass ratio of 1:80 and stirred at room temperature overnight to obtain the BSCS@PHY.

### 4.3 Evaluation of the Photothermal Effect of BSC and BSCS@PHY

To evaluate photothermal performance, aqueous solutions of BSC/BSCS@PHY at varying concentrations (25, 50, 100, and 200 µg mL^−1^) were exposed to an 808 nm laser (1 W cm^−2^) for 10 min. Temperature evolution was tracked by infrared thermography, with DI water serving as a control.

To examine the effect of laser power density, a 100 µg mL^−1^ aqueous solution of BSC/BSCS@PHY nanoparticles was irradiated with an 808 nm laser at varying power densities (0.5, 1, 1.5, and 2 W cm^−2^) for 10 min. Temperature changes were tracked by infrared thermography, with DI water serving as a control.

For photothermal stability testing, 1 mL of a 100 µg mL^−1^ BSC/BSCS@PHY aqueous solution was placed in a colorless, transparent square cuvette and irradiated with an 808 nm laser at 1 W cm^−2^ for 10 min. The laser was then turned off, allowing the solution to cool naturally to room temperature. This on‐off cycle was repeated four times, and temperature changes throughout the process were measured using infrared thermography.

### 4.4 Evaluation of Toxic ROS Generation Capacity

•OH generation by the nanoparticles was quantified using the 3,3′,5,5′‐tetramethylbenzidine (TMB) assay. In this assay, colorless TMB is oxidized to blue‐green oxidized TMB (ox‐TMB) with an absorption maximum near 650 nm. To assess ROS generation, 50 µg mL^−1^ of BSC, BSC@PH, BSCS@PH, and BSCS@PHY were incubated with 0.2 mM TMB in buffer solutions adjusted to pH 6.5 or pH 7.4, with or without the addition of 2 mM H_2_O_2_, at room temperature for 3 h. Absorbance at ∼650 nm was used as the proxy for •OH production. Control groups included TMB alone and TMB with 2 mM H_2_O_2_.

Further, the influence of temperature on ROS generation was investigated by incubating 50 µg mL^−1^ of BSC, BSC@PH, BSCS@PH, or BSCS@PHY with 0.2 mM TMB at either room temperature or 37 °C for 30 min in pH 6.5 buffer, with or without 2 mM H_2_O_2_. After incubation, the samples were detected using a UV‐Vis spectrophotometer to assess •OH radical generation. TMB alone and TMB+2 mM H_2_O_2_ were used as controls.

### 4.5 GSH Consumption Assay

To investigate the ability of nanoparticles to deplete glutathione (GSH), an assay utilizing Ellman's reagent [5,5′‐dithiobis(2‐nitrobenzoic acid), DTNB] was performed. In this assay, GSH reduces DTNB to a yellow compound with an absorption peak at 412 nm. At pH 6.5, BS and BSC nanoparticles at a series of concentrations (20, 40, and 60 µg mL^−1^) were incubated with GSH (0.2 mM) for 2 h at room temperature. Following incubation, DTNB (0.2 mM) was introduced, and absorbance at 412 nm was recorded using a microplate reader. Control groups included DTNB alone (negative control) and DTNB + GSH (positive control).

Similarly, BSC and BSCS@PHY were incubated at varying concentrations (5, 10, 20, 40, 60, and 80 µg mL^−1^) with GSH (0.2 mM) at room temperature in a pH 6.5 buffer for 2 h. Afterward, DTNB (0.2 mM) was added, and the absorbance at 412 nm was recorded. DTNB alone and DTNB + GSH served as negative and positive controls, respectively.

### 4.6 Cell Lines and Animals

4T1 and Luc‐4T1 murine breast cancer lines were purchased from the American Type Culture Collection (ATCC). Parental 4T1 and luciferase‐expressing Luc‐4T1 cells were cultured in RPMI‐1640 supplemented with 10% fetal bovine serum (FBS) and 1% penicillin/streptomycin (P/S). Cells were maintained at 37°C in a humidified 5% CO_2_ atmosphere and subcultured two to three times per week upon reaching 90–100% confluence. Bone marrow‐derived dendritic cells (BMDCs) were prepared from 6‐8‐week‐old BALB/c mice. Tibiae and femurs were dissected, placed on ice, and rinsed in a sterile dish. Under aseptic conditions, the epiphyses were removed, and the marrow cavity was flushed with culture medium using a sterile syringe to expel marrow. After red blood cell lysis, the cell suspension was washed and seeded in RPMI‐1640 containing 10% FBS, 1% P/S, 20 ng mL^−1^ GM‐CSF, and 10 ng mL^−1^ IL‐4, and cultured for 7 days to induce BMDC differentiation. Cells were maintained at 37°C in a humidified 5% CO_2_ atmosphere. Healthy 5‐6‐week‐old female BALB/c mice were sourced from the Guangdong Medical Laboratory Animal Center (Guangdong, China). All in vivo experiments were conducted in accordance with the Guidelines for the Care and Use of Laboratory Animals and were approved by the Laboratory Animal Ethics Committee of Jinan University (IACUC‐20210915‐02). The maximum permissible tumor size, as set by the ethics committee or institutional review board, was 2000 mm^3^, and no experimental groups exceeded this limit.

### 4.7 Investigation of Cellular Uptake

The cellular uptake of nanoparticles was assessed using organic dyes. ICG was added to aqueous solutions of BSC and BSCS@PH at a weight ratio of 1:10 and stirred for 4 h. The mixtures were then centrifuged and washed twice with DI water to obtain ICG‐labeled BSC and ICG‐labeled BSCS@PH. For BSCS@PHY, ICG‐labeled BSCS@PH was further incubated with YCB at a weight ratio of 80:1 and stirred overnight to obtain ICG‐labeled BSCS@PHY. The same procedure was applied to prepare RBITC‐labeled BSC, BSCS@PH, and BSCS@PHY, replacing ICG with RBITC.

To assess cellular uptake, 4T1 cells were plated in 24‐well plates and allowed to adhere overnight. Cells were then incubated for 6 h with ICG‐labeled BSC, BSCS@PH, or BSCS@PHY. After exposure, the medium was aspirated, cells were rinsed twice with PBS, harvested, and analyzed by flow cytometer (BD, FACSCanto).

Additionally, 4T1 cells were placed in a confocal dish and allowed to adhere overnight. The next day, cells were treated with RBITC‐labeled BSC, BSCS@PH, or BSCS@PHY for 6 h, followed by two PBS washes. Lysosomes were then labeled with LysoTracker Green for 1.5 h, rinsed twice with PBS, counterstained with Hoechst 33342 for approximately 20 min, rinsed twice again, and imaged on a confocal laser scanning microscope (CLSM, Zeiss LSM‐800).

### 4.8 Evaluation of Cytotoxicity

To evaluate the therapeutic efficacy of BSCS@PHY in cancer treatment, cell viability was quantified by the Alamar Blue assay. Particularly, 4T1 cells were seeded in 96‐well plates (1 × 10^4^ cells/well) and allowed to adhere overnight. The next day, medium was replaced with fresh medium containing BSC, BSCS@PH, or BSCS@PHY at 1000, 500, 250, 125, 62.5, 31.25, 15.63, 7.815, or 3.908 µg mL^−1^. After 5 h, wells were either exposed to NIR irradiation (1 W cm^−2^, 10 min) or kept as non‐irradiated controls, followed by a further 19 h incubation. Media were then exchanged for fresh medium supplemented with 10% Alamar Blue working solution, and cells were incubated for approximately 1.5 h. Fluorescence emission at 590 nm was measured using a microplate reader, and viability was calculated relative to untreated controls.

### 4.9 Live/Dead Fluorescent Staining

4T1 cells were seeded in 24‐well plates (1×10^5^ cells/well) and allowed to adhere overnight. Cells were then treated with BSC, BSCS@PH, or BSCS@PHY (100 µg mL^−1^) for 5 h. Where indicated, cultures were irradiated with an NIR laser (1 W cm^−2^, 10 min); parallel wells were left non‐irradiated. Media were replaced with fresh complete medium, and cells were incubated for an additional 2 h. After a PBS rinse, cells were stained with a Live/Dead viability kit (∼30 min) to distinguish Calcein‐AM‐positive (live) from propidium iodide (PI)‐positive (dead) cells, rinsed once with PBS, and imaged on a fluorescence microscope (Carl Zeiss, Observer Z1). Excitation wavelengths: 488 nm (Calcein‐AM) and 532 nm (PI).

### 4.10 Apoptosis Assay

For quantitative analysis of photoactivation‐induced apoptosis, 4T1 cells were seeded in 24‐well plates at a density of 1 × 10^5^ cells per well and allowed to adhere overnight. Cells were then incubated with BSC, BSCS@PH, or BSCS@PHY (150 µg mL^−^^1^) for 5 h before NIR irradiation, based on the cellular uptake assessment described above. The cells were irradiated with an NIR laser at 1 W cm^−^^2^ for 10 min or kept without irradiation. After irradiation, the medium was replaced with fresh medium, and the cells were further incubated for 2 h before apoptosis analysis to evaluate early apoptosis‐related changes induced by photoactivation. Cells were then collected, washed with PBS, and resuspended in 100 µL Annexin‐binding buffer. For apoptosis staining, 5 µL Annexin V‐FITC and 10 µL PI were added to the cell suspension and incubated for 15 min in the dark. The staining reaction was terminated by adding 400 µL Annexin‐binding buffer. Apoptosis was quantified by flow cytometry (BD, FACSCanto).

### 4.11 Evaluation of Intracellular ROS Generation Capacity in Vitro

Intracellular ROS levels were evaluated using the cell‐permeable fluorogenic probe 2′,7′‐dichlorodihydrofluorescein diacetate (DCFH‐DA) [100]. After entering cells, DCFH‐DA is deacetylated by intracellular esterases to nonfluorescent DCFH, which can be oxidized by ROS to fluorescent dichlorofluorescein (DCF), enabling detection of intracellular ROS levels. Briefly, 4T1 cells were seeded in 24‐well plates at a density of 1 × 10^5^ cells per well and allowed to adhere overnight. The next day, cells were incubated with the indicated formulations for 5 h before NIR irradiation, with this incubation time selected based on the cellular uptake assessment described above. The groups were set up as follows: PBS, BSC, BSCS@PH, BSCS@PHY, PBS + H_2_O_2_, BSC + H_2_O_2_, BSCS@PH + H_2_O_2_, BSCS@PHY + H_2_O_2_, PBS + Laser, BSC + Laser, BSCS@PH + Laser, BSCS@PHY + Laser, PBS + H_2_O_2_ + Laser, BSC + H_2_O_2_ + Laser, BSCS@PH + H_2_O_2_ + Laser, and BSCS@PHY + H_2_O_2_ + Laser. The formulation concentration was 100 µg mL^−^^1^, and the H_2_O_2_ concentration was 100 µM. Cells were then exposed to an NIR laser at 1 W cm^−^^2^ for 10 min or kept without irradiation. After irradiation, the medium was replaced with fresh medium, and cells were further incubated for 2 h to allow early photoactivation‐induced oxidative responses to develop before ROS detection. After washing once with PBS, cells were incubated with 200 µL fresh medium containing 10 µM DCFH‐DA for 20 min. The medium was then removed, and the cells were rinsed twice with PBS. Intracellular ROS levels were analyzed by fluorescence microscopy or flow cytometry.

### 4.12 In Vitro GSH Depletion Assessment

Intracellular GSH levels were quantified using a GSH and GSSG assay kit in accordance with the manufacturer's protocol. In brief, 4T1 cells were plated in 24‐well plates (1 × 10^5^ cells/well) and allowed to adhere overnight. Cells were then incubated with BSC, BSCS@PH, or BSCS@PHY (100 µg mL^−1^) for 5 h. Following the treatment, the cells were washed twice with PBS and collected for GSH and GSSG analysis.

### 4.13 Assessment of DNA Damage in Vitro

4T1 cells were placed in 24‐well plates at a density of 1 × 10^5^ cells per well and allowed to adhere overnight. Cells were incubated with BSC, BSCS@PH, or BSCS@PHY (100 µg mL^−^^1^) for 5 h before NIR irradiation, based on the cellular uptake assessment described above. The cells were then irradiated with an NIR laser at 1 W cm^−^^2^ for 10 min or kept without irradiation. After irradiation, the medium was replaced with fresh medium, and the cells were further incubated for 2 h before γ‐H2AX staining to capture early photoactivation‐induced DNA damage. γ‐H2AX immunofluorescence staining was performed using a DNA damage detection kit according to the manufacturer's instructions. The cells were then rinsed with PBS and imaged under a fluorescence microscope (Carl Zeiss, Observer Z1).

### 4.14 Evaluation of ICD Induction Efficacy

1. Assessment of calreticulin (CRT) cell‐surface exposure: 4T1 cells were seeded in 24‐well plates at a density of 1 × 10^5^ cells per well and allowed to adhere overnight. The next day, cells were treated with BSC, BSCS@PH, or BSCS@PHY at a concentration of 100 µg mL^−^^1^ for 5 h before NIR irradiation, based on the cellular uptake assessment described above. The cells were then irradiated with an 808 nm NIR laser at 1 W cm^−^^2^ for 10 min or left unirradiated. For flow cytometry analysis, the medium was replaced with fresh growth medium, and cells were further incubated for 2 h before staining to evaluate early membrane CRT exposure. Cells were then collected, rinsed with PBS, fixed with 4% paraformaldehyde for 10 min, washed, and blocked with 0.5% BSA for 30 min without permeabilization. Samples were incubated overnight at 4°C with a primary anti‐CRT antibody, washed three times with PBS, labeled with an Alexa Fluor 488‐conjugated secondary antibody, and analyzed by flow cytometry. For fluorescence microscopy analysis, after NIR irradiation or no irradiation, the medium was replaced with fresh growth medium, and cells were cultured for an additional 12 h. Cells were then fixed, blocked, and immunostained for CRT without permeabilization as described above. Nuclei were counterstained with DAPI, and images were acquired by fluorescence microscopy.
2. Evaluation of HMGB1 Release: 4T1 cells were seeded in 24‐well plates (1×10^5^ cells/well) and allowed to adhere overnight. Cells were then incubated with BSC, BSCS@PH, or BSCS@PHY (100 µg mL^−1^) for 5 h and then either irradiated with an 808 nm NIR laser (1 W cm^−2^, 10 min) or left unirradiated. For HMGB1 detection using an ELISA kit, the medium was refreshed with the growth medium and maintained overnight. Afterward, the supernatant was harvested for analysis of extracellular HMGB1 contents. For fluorescence microscopy analysis, cells were replenished with fresh growth medium and incubated for an additional 4 h. They were then fixed with 4% paraformaldehyde for 10 min, permeabilized with 0.2% Triton X‐100 for 10 min, and blocked with 0.5% BSA for 30 min. Samples were incubated overnight at 4°C with an anti‐HMGB1 primary antibody, washed three times with PBS, and labeled with Alexa Fluor 488‐conjugated secondary antibody. For imaging, nuclei were counterstained with DAPI and examined by fluorescence microscopy. For flow‐cytometric quantification of intracellular HMGB1, cells were detached after secondary labeling, collected, and analyzed on a flow cytometer.
3. Evaluation of ATP Release: 4T1 cells were seeded in 24‐well plates (1×10^5^ cells/well) and allowed to adhere overnight. Cells were treated with BSC, BSCS@PH, or BSCS@PHY (100 µg mL^−1^) for 5 h and exposed to an NIR laser (1 W cm^−2^, 10 min) or left untreated. Afterward, the medium was replenished with a fresh growth medium and maintained overnight. The supernatant was then harvested for extracellular ATP concentration analysis using an ATP determination kit.

### 4.15 Immunomodulatory Effects of the BSCS@PHY Cascade Nanoplatform on Dendritic Cells

BMDCs were incubated for 24 h with various formulations, including LPS (1 µg mL^−1^), yeast cell walls (YCW, 0.5 µg mL^−1^), and BSC/BSCS@PH/BSCS@PHY (40 µg mL^−1^ each). After incubation, the cells were harvested, washed, and analyzed by flow cytometry to assess surface markers indicative of BMDC maturation.

4T1 cells were placed in 24‐well plates (1×10^5^ cells/well) and allowed to adhere overnight. The cells were then treated with PBS, SCH442416, BSC, BSCS@PH, or BSCS@PHY (nanoparticle concentration:100 µg mL^−1^; SCH442416 concentration: 25 µg mL^−1^) for 5 h, followed by irradiation with an 808 nm NIR laser (1 W cm^−2^, 10 min). After an overnight incubation, the supernatants were collected and centrifuged to remove cell debris, resulting in the following treatment groups: (PBS+Laser)‐treated supernatants, (SCH+Laser)‐treated supernatants, (BSC+Laser)‐treated supernatants, (BSCS@PH+Laser)‐treated supernatants, and (BSCS@PHY+Laser)‐treated supernatants.

The (PBS+Laser)‐treated and (SCH+Laser)‐treated supernatants were then separately introduced to immature BMDCs derived from the bone marrow of BALB/c mice. LPS (working concentration: 1 µg mL^−1^) and NECA (working concentration: 5 µM) were subsequently introduced, and the cells were incubated for 24 h. Additionally, the (BSC+Laser)‐treated supernatants, (BSCS@PH+Laser)‐treated supernatants, and (BSCS@PHY+Laser)‐treated supernatants were separately added to BMDCs, followed by the introduction of NECA (working concentration: 5 µM). Cells were then incubated for 24 h. Moreover, LPS (working concentration: 1 µg mL^−1^) was added to BMDCs and incubated for 24 h to serve as the positive control group. Following this, BMDCs were harvested for flow cytometry analysis to evaluate their maturation status; Additionally, treated cells were collected for western blot analysis. Cells were rinsed once with PBS and lysed in RIPA buffer supplemented with 1% phosphatase and 1% protease inhibitor cocktails. After 30 min on ice, lysates were clarified by centrifugation (12 000 rpm, 15 min, 4 °C). The supernatant was collected, and protein concentration was determined using a BCA assay. Equal amounts of protein (25 µg per lane) were separated on 12% SDS‐PAGE and transferred to PVDF membranes using a Tris‐glycine‐methanol buffer (20 mM Tris, 150 mM glycine, 20% methanol). Membranes were blocked in 5% non‐fat milk in TBST at room temperature for 1.5 h, then incubated overnight at 4°C with anti‐A2AR primary antibody (1:1000). After three 10‐min TBST washes, membranes were incubated with HRP‐conjugated anti‐rabbit IgG (1:1000) for 1 h at room temperature. Signals were developed by chemiluminescence and captured on a digital imager (Amersham Imager 600, GE). For loading normalization, the same membranes were washed, re‐blocked, and reprobed with anti‐GAPDH (1:10000) followed by the appropriate HRP‐secondary, and GAPDH bands were acquired under identical imaging settings.

### 4.16 Biodistribution

To establish the 4T1 tumor model, mice were inoculated subcutaneously in the right flank with 1.5×10^6^ 4T1 cells. For in vivo biodistribution and tumor accumulation, tumor‐bearing mice (tumor volume ∼100‐150 mm^3^) received an intravenous dose of ICG‐labeled BSCS@PHY (20 mg kg^−1^). At 72 h post‐injection, tumors and major organs (heart, liver, spleen, lung, and kidney) were excised and imaged ex vivo using an IVIS system (PerkinElmer IVIS Lumina Series III). Fluorescence signals from each tissue were quantified with the instrument software to assess nanoparticle biodistribution and tumor uptake.

### 4.17 Evaluation of in Vivo Photothermal Properties

In vivo photothermal performance was assessed in 4T1 tumor‐bearing mice (tumor volume ∼100‐150 mm^3^). Animals were randomized to receive a single intravenous dose (20 mg kg^−1^) of either BSC@PH or BSCS@PHY. At 36 h post‐injection, tumor sites were irradiated with an NIR laser (1 W cm^−2^, 10 min), and surface temperature dynamics were recorded by infrared thermographic imaging.

### 4.18 Assessment of Antitumor Efficacy in Vivo

In 5‐6‐week‐old female BALB/c mice, unilateral 4T1 tumors were established by subcutaneous injection of 1.5×10^6^ 4T1 cells into the right flank. When tumors reached ∼100‐150 mm^3^, animals were randomly assigned to ten groups (n = 7 per group): PBS (G1), anti‐PD‐L1 (G2, α‐PDL1), SCH442416 (G3), BSC@PH (G4), BSC@PH+Laser (G5, BSC@PH+L), BSCS@PH (G6), BSCS@PH+Laser (G7, BSCS@PH+L), BSCS@PHY (G8), BSCS@PHY+Laser (G9, BSCS@PHY+L), BSCS@PHY+Laser+anti‐PD‐L1 (G10, BSCS@PHY+L+α‐PDL1). SCH442416 (5 mg kg^−1^) and various nanoformulations (20 mg kg^−1^) were administered intravenously to the mice on days 1, 5, 7, 9, 13, and 15. The tumor sites of mice in groups G5, G7, G9, and G10 were treated with a 10‐min NIR laser at 1 W cm^−2^ on days 3 (approximately 36 h after the day 1 intravenous injection) and day 11 (approximately 36 h after the day 9 intravenous injection). Additionally, the mice received intraperitoneal injections of anti‐PD‐L1 antibody (100 µg/mouse) on days 4, 8, 12, and 16. Tumor burden and body mass were assessed on alternate days using a digital caliper and an analytical balance, respectively. Tumor volume (V) was estimated as V = (L × W^2^)/2, where L is the longest tumor diameter, and W is the shortest diameter. At the study endpoint, blood was collected for clinical chemistry. Mice were then euthanized, and major organs, TDLNs, and tumors were carefully excised. Tumors were weighed and photographed ex vivo. Representative portions of major organs and tumor tissues were fixed in 4% paraformaldehyde and processed for H&E staining. For immunofluorescence, TDLNs and tumor sections were likewise fixed and prepared according to the supplier's instructions. Stained slides were imaged on a whole‐slide scanner (Olympus VS200) or examined by fluorescence microscopy. Fresh spleen, TDLN, and tumor samples were immediately converted to single‐cell suspensions via mechanical dissociation with enzyme digestion as appropriate, red‐blood‐cell (RBC) lysis, and filtration. Before acquisition, cells were blocked with anti‐CD16/32 to prevent Fc‐mediated binding, stained with the indicated fluorophore‐conjugated antibodies per the manufacturer's protocol, and analyzed by flow cytometry.

### 4.19 Evaluation of Distant‐Tumor Inhibition

A bilateral 4T1 model was used (Figure 8A). Female BALB/c mice (5‐6 weeks) were inoculated subcutaneously with 1.5×10^6^ 4T1 cells in the right flank (primary tumors). Five days later, a second inoculation of 1.5×10^6^ 4T1 cells was given in the contralateral (left) flank to establish a distant tumor. When tumors reached ∼100‐150 mm^3^, mice were randomized into three groups (n = 4): PBS, BSCS@PHY+Laser (BL), and BSCS@PHY+Laser+anti‐PD‐L1 (BLP). Dosing and irradiation schedules matched those in the unilateral model. Tumor dimensions were measured every other day with a caliper, and volumes were calculated as (L × W^2^)/2, where L and W denote the longest and shortest diameters, respectively. Five days after the final treatment, mice were euthanized and the spleen, TDLNs, and tumors were collected. Tumors were photographed ex vivo, and freshly isolated spleen/TDLN/tumor samples were prepared as single‐cell suspensions using standard mechanical dissociation, enzymatic digestion as appropriate, RBC lysis, and filtration. Before acquisition, cells were Fc‐blocked with anti‐CD16/32 and stained with the indicated fluorophore‐conjugated antibodies according to the manufacturer's instructions for flow‐cytometry.

### 4.20 Assessment of Lung‐Metastasis Inhibition

A lung‐metastasis model was established (Figure 9A). Female BALB/c mice (5‐6 weeks) first received a subcutaneous inoculation of 1.5×10^6^ 4T1 cells in the right flank to generate a primary tumor. When tumors reached ∼100‐150 mm^3^, Luc‐4T1 cells (2×10^5^) were injected intravenously (tail vein) 1 day before treatment initiation to seed pulmonary metastases. Mice were then randomized into three cohorts (n = 5): PBS, BSCS@PHY+Laser (BL), and BSCS@PHY+Laser+anti‐PD‐L1 (BLP). Dosing and irradiation schedules matched those used in the unilateral model. To monitor lung metastasis, mice received intraperitoneal D‐luciferin (potassium salt, 150 mg kg^−1^) on days 4, 11, 18, 22, and 25 after treatment start, followed by bioluminescence imaging on an IVIS system under fixed acquisition settings. Eleven days after the final treatment, mice were euthanized, and the spleens, TDLNs, and lungs were harvested. Lungs were photographed ex vivo and imaged by bioluminescence. Fresh spleen, TDLN, and lung tissues were immediately processed into single‐cell suspensions using standard mechanical dissociation, enzymatic digestion as appropriate, RBC lysis, and filtration. Cells were then Fc‐blocked with anti‐CD16/32 and stained with the indicated fluorophore‐conjugated antibodies per the manufacturer's protocol prior to flow‐cytometric analysis.

### 4.21 Evaluation of Inhibitory Effects on Rechallenged 4T1 Tumors

Female BALB/c mice (5–6 weeks) bearing unilateral 4T1 tumors were established by subcutaneous inoculation of 1.5 × 10^6^ 4T1 cells into the right flank. When tumors reached ∼100‐150 mm^3^, mice were randomly assigned to PBS, BSCS@PHY + Laser, or BSCS@PHY + Laser + anti‐PD‐L1. Dosing and irradiation schedules matched those used in the unilateral model. At day 110 after the last treatment, surviving mice (total n = 8) were rechallenged by subcutaneous injection of 1.5 × 10^6^ 4T1 cells into the right flank; age‐matched naïve BALB/c females received the same inoculum as a control cohort. Tumor size was measured every other day with digital calipers, and volume was calculated as (L × W^2^)/2, where L and W denote the longest and shortest tumor diameters, respectively.

### 4.22 Quantitative Proteomic Analysis

Tumor tissues were collected from mice in the PBS, BSCS@PHY+Laser, and BSCS@PHY+Laser+anti‐PD‐L1 groups (n = 3). Each specimen was lysed in RIPA buffer (Thermo Fisher, 89901) using a bead‐mill homogenizer (Bead Ruptor Elite, OMNI, USA) on ice/4 °C. Lysates were cleared by centrifugation (13000 rpm, 10 min, 4 °C), and total protein was quantified with the Pierce BCA Protein Assay Kit (Thermo Fisher, 23227). Equal protein masses were resolved by SDS‐PAGE and subjected to in‐gel digestion as described [101]. Briefly, Coomassie Brilliant Blue‐stained bands were excised, destained, and digested in‐gel with sequencing‐grade trypsin (Sigma‐Aldrich, V5280). The resulting peptides were desalted on C18 cartridges, dried in a vacuum concentrator, and reconstituted in 0.1% formic acid. Peptide mixtures were analyzed by LC‐MS/MS on an Orbitrap Ascend mass spectrometer coupled to a Vanquish Neo UHPLC (Thermo Fisher, USA).

Data‐dependent acquisition (DDA) RAW files were processed in MaxQuant (v2.6.4.0) using default settings unless noted. Precursor (MS1) and fragment (MS/MS) mass tolerances were set to 4.5 and 20 ppm, respectively. Searches were performed against the UniProtKB Mus musculus database (taxonomy 10090; release 2024‐11‐07) using a concatenated target–decoy (reversed) strategy to control false discoveries. Trypsin was specified as the protease with up to two missed cleavages. Fixed and variable modifications were carbamidomethyl (C) and acetyl (protein N‐term) / oxidation (M), respectively. The options “Second peptides,” “Match between runs”, and “Dependent peptides” were enabled. Peptides had to be ≥ 7 amino acids. False discovery rate (FDR) was controlled at 1% at the PSM/peptide and protein levels (with site‐level FDR applied where relevant); no additional fixed score cutoffs were imposed beyond FDR control. Label‐free quantification (MaxLFQ) was used to estimate protein abundances.

Differential protein expression was evaluated on the quantitative dataset; proteins with a fold change ≥ 1.5 and *p* ≤ 0.05 were designated as differentially expressed proteins (DEPs). Volcano plots (log_2_(fold change) vs −log_10_(*p*‐value)) were generated to visualize DEP distributions.

### 4.23 GO and KEGG Enrichment Analysis

Functional enrichment for Gene Ontology (GO) terms and Kyoto Encyclopedia of Genes and Genomes (KEGG) pathways was carried out using the DAVID database. For both analyses, the genes mapped from the differentially expressed proteins (DEPs) were used as the input set, while all genes corresponding to the identified proteome served as the background, providing a proteome‐derived enrichment context rather than a whole‐genome reference. GO enrichment was summarized across biological process (BP), cellular component (CC), and molecular function (MF), providing functional insights into the proteomic changes induced by the treatment conditions. KEGG pathway enrichment was conducted to systematically identify biological pathways related to the treatment‐induced proteomic changes, including metabolism, genetic information, environmental information processing, cellular processes, organismal systems, human diseases, and drug development pathways. Enrichment results were visualized using bar plots for GO categories, with enrichment scores on the x‐axis for BP, CC, and MF terms. KEGG pathways were displayed using bubble plots, in which enrichment score, gene count, and −log_10_(*p*‐value) were mapped to the x‐axis, bubble size, and bubble color, respectively. For visualization, pathways with nominal *p* ≤ 0.05 were ordered by their *p*‐values, and the top ten terms were shown. FDR‐adjusted *p*‐values were also calculated and reported as a reference. In interpreting the enrichment results, we focused on functional patterns that recurred across GO terms, KEGG pathways, and the underlying protein‐level changes.

### 4.24 Statistical Analysis

Data are presented as mean ± standard deviation (SD), with the sample size (n) indicated in the corresponding figure legends. Data were analyzed without transformation unless otherwise indicated in the corresponding figures, figure legends, or Methods section. For normalized data, the normalization method is described in the corresponding figure legends or Methods section. Comparisons between two groups were performed using unpaired, two‐tailed Student's t‐tests. For comparisons among multiple groups, one‐way ANOVA followed by Tukey's multiple‐comparisons test was used. For experiments involving two independent variables, two‐way ANOVA followed by Tukey's multiple‐comparisons test was performed. Statistical significance was defined as *P* < 0.05. Significance levels are indicated as ^*^ *P* < 0.05, ^**^ *P* < 0.01, ^***^ *P* < 0.001, and ^****^ *P* < 0.0001; n.s., not significant. Statistical analyses were performed using GraphPad Prism 10.

## Author Contributions

**Xiangting Yi**: conceptualization, investigation, methodology, validation, visualization, formal analysis, data curation, writing – original draft. **Hanlou Yang**: conceptualization, investigation, methodology, writing – original draft, validation, visualization, formal analysis, data curation. **Xiaoyu Huang**: data curation, formal analysis, methodology, investigation, validation. **Shiying Zhou**: conceptualization, investigation, methodology, validation, visualization, formal analysis. **Pengfei Wu**: investigation, methodology, visualization, formal analysis, data curation. **Mingqiang Li**: conceptualization, methodology, investigation, supervision, writing – review and editing, validation. **Min Han**: conceptualization, investigation, methodology, validation, visualization, formal analysis, data curation. **Jie Zhou**: conceptualization, methodology, investigation, supervision, writing – review and editing, validation. **Yiling Zhong**: conceptualization, investigation, funding acquisition, writing – original draft, writing – review and editing, project administration, methodology, supervision, visualization, validation, formal analysis. **Zishan Chen**: conceptualization, investigation, methodology, validation, visualization, formal analysis, data curation. **Junting Huang**: investigation, methodology, validation, formal analysis, data curation. **Nan Ma**: conceptualization, writing – review and editing, investigation, supervision, methodology, validation. **Zunde Liao**: conceptualization, investigation, methodology, validation, visualization, formal analysis, data curation.

## Conflicts of Interest

The authors declare no conflicts of interest.

## Supporting information




**Supporting File**: advs76303‐sup‐0001‐SuppMat.docx.

## Data Availability

The data that support the findings of this study are available from the corresponding author upon reasonable request.
