# Supplementary material for: Material‐Encoded Synchronization of Immunogenic Cell Death With Adenosine A2A Receptor Blockade Reprograms the Tumor Microenvironment
Source: Adv Sci (Weinh). 2026 Jun 28;13(52):e76303. doi: 10.1002/advs.76303 (PMC13336662; doi:10.1002/advs.76303)
Supplement: Supplementary file 1 [file ADVS-13-e76303-s001.docx]

**Supporting Information**

**Material-Encoded Synchronization of Immunogenic Cell Death with Adenosine A2A Receptor Blockade Reprograms the Tumor Microenvironment**

*Xiangting Yi**^#^, Hanlou Yang^#^, Junting Huang, Pengfei Wu, Shiying Zhou, Zunde Liao, Min Han, Zishan Chen, Xiaoyu Huang, Nan Ma^*^, Jie Zhou^*^,* *Mingqiang Li^*^, Yiling Zhong^*^*

X. T. Yi, H. L. Yang, J. T. Huang, P. F. Wu, S. Y. Zhou, Z. D. Liao, M. Han, Z. S Chen, X. Y. Huang, N. Ma, Y. L. Zhong

State Key Laboratory of Bioactive Molecules and Druggability Assessment, Guangdong Basic Research Center of Excellence for Natural Bioactive Molecules and Discovery of Innovative Drugs, College of Pharmacy, Jinan University, Guangzhou, Guangdong 511443, P. R. China

E-mail: nanma927@126.com (N. Ma), yilingzhong@jnu.edu.cn (Y. L. Zhong)

J. Zhou

Department of Breast Surgery, Affiliated Cancer Hospital and Institute of Guangzhou Medical University, Guangzhou 510095, P. R. China

E-mail: [sunnysmile@gzhmu.edu.cn](mailto:sunnysmile@gzhmu.edu.cn) (J. Zhou)

M. Q. Li

Laboratory of Biomaterials and Translational Medicine, Center for Nanomedicine, The Third Affiliated Hospital, Sun Yat-sen University, Guangzhou 510630, P. R. China

E-mail: limq567@mail.sysu.edu.cn (M. Q. Li)

*^#^*These authors contributed equally to this work

**Figure S1.** Survey XPS spectra of BSC.

**Figure** **S2.** The UV-vis-NIR spectra of different formulations.

**Figure** **S3.** TEM image of BSCS@PHY.

**Figure** **S4.** Elemental distribution mapping images of BSCS@PHY.

**Figure** **S5.** Digital photographs of aqueous solutions containing various formulations.

**Figure** **S6.** (A) Temperature-time curves illustrating the photothermal effect of BSCS@PHY in aqueous solution at various concentrations under NIR laser irradiation (1 W cm^-2^, 10 minutes). (B) Temperature-time curves demonstrating the photothermal response of BSCS@PHY at a concentration of 100 μg mL^-1^ in aqueous solution, exposed to multiple NIR laser power densities. (C) Real-time temperature curves depicting the photothermal stability of BSCS@PHY in aqueous solution during four on/off cycles of NIR laser exposure.

**Figure** **S7.** Characterization of ROS generation. (A, B) Digital photographs of the various samples incubated for 3 hours at room temperature under pH 7.4 (A) and pH 6.5 (B). Concentrations used: H_2_O_2_ at 2 mM, TMB at 0.2 mM, and BSC-based formulations at 50 μg mL^-1^.

**Figure** **S8.** Characterization of ROS generation. (A-D) UV–Vis absorption spectra (A, C) and corresponding photographs (B, D) of TMB alone, TMB with H_2_O_2_, TMB with BSC-based formulations, and TMB with both H_2_O_2_ and BSC-based formulations at pH 6.5 after 30 min of incubation at room temperature (A, B) and 37 °C (C, D). Final concentrations were as follows: H_2_O_2_ (2 mM), TMB (0.2 mM), and BSC-based formulations (50 μg mL^-1^).

**Figure S9.** Assessment of GSH depletion by BS and BSC at varying concentrations, using DTNB (0.2 mM) as a trapping agent (GSH: 0.2 mM). UV-vis absorption spectra depicting GSH alone, GSH with DTNB, and GSH in combination with DTNB and different concentrations of BS or BSC.

**Figure** **S10.** (A) Flow cytometry histograms showing the cellular uptake of ICG-labeled BSC, ICG-labeled BSCS@PH and ICG-labeled BSCS@PHY in 4T1 cells. (B) Quantitative flow cytometry analysis corresponding to Figure S10A, showing the cellular uptake of ICG-labeled BSC, ICG-labeled BSCS@PH and ICG-labeled BSCS@PHY in 4T1 cells.

**Figure** **S11.** (A) Viability of 4T1 cells after treatment with various formulations at different concentrations. (B) Viability of 4T1 cells following treatment with various formulations at different concentrations combined with laser irradiation. Data are presented as mean ± SD (n = 3 per group).

**Figure** **S12.** (A) Total GSH levels in 4T1 cells after different treatments, measured using a GSH and GSSH assay kit. (B) GSH levels in 4T1 cells after different treatments, measured using a GSH and GSSH assay kit. Data are presented as mean ± SD (n = 3 per group). Statistical significance across groups was assessed using one-way ANOVA and Tukey’s posttest. Significance levels are indicated as follows: ***P* < 0.01, ****P* < 0.001.

**Figure** **S13.** Quantitative analysis of γ-H2AX, as determined by fluorescent imaging. Statistical significance across groups was assessed using one-way ANOVA and Tukey’s posttest. Significance levels are indicated as follows: *****P* < 0.0001.

**Figure** **S14.** (A) Quantitative analysis of CRT expression on cell surfaces, as determined by fluorescent imaging. (B) Representative flow cytometry analysis showing CRT surface exposure on tumor cells following different treatments. (C) Quantification of HMGB1 fluorescent intensity in 4T1 cells following various treatments, as determined by fluorescent imaging. (D) Flow cytometric quantification of HMGB1 expression in 4T1 cells under various treatments. Data are presented as mean ± SD (n = 3 per group). Statistical significance across groups was assessed using one-way ANOVA and Tukey’s posttest. Significance levels are indicated as follows: **P* < 0.05, ***P* < 0.01, *****P* < 0.0001.

**Figure** **S15.** Representative Western blot images showing A2AR and GAPDH protein levels in BMDCs from different treatment groups.

**Figure S16.** Individual tumor growth trajectories in mice treated with various regimens (n = 7). The arrow indicates the day of laser administration.

**Figure** **S17.** Representative digital images of mice receiving diverse treatments on days 0, 8, and 16.

**Figure** **S18.** (A) Individual tumor growth trajectories in mice treated with various regiments (n = 8). (B) Survival curves of mice after various treatments (n=8).

**Figure** **S19.** (A) The H&E images of tumors and different organs from mice subjected to various treatments. (B) Changes in body weight of mice treated with different formulations (n = 7). (C) Endpoint blood biochemistry analysis of mice from different treatment groups (n=3).

**Figure** **S20.** (A) Representative immunofluorescence images showing CRT exposure in tumor tissues from mice undergoing different therapeutic interventions. (B) Representative immunofluorescence images showing A2AR expression in TDLN tissues from mice undergoing different therapeutic interventions. (C) Representative immunofluorescence images depicting CD8⁺ T cell infiltration in tumor tissues from mice subjected to different therapeutic interventions. (D) Representative immunofluorescence images showing CD4^+^ T cell expression in TDLNs from mice receiving different treatments.

**Figure** **S21.** (A) Individual primary tumor growth curves of mice after different treatments (n=4). The arrow indicates the day of laser administration. (B) Individual distant tumor growth curves of mice after different treatments (n=4). (C) Weights of excised primary tumor tissues from mice following different treatment regimens (n = 4). (D) Weights of excised distant tumor tissues from mice following different treatment regimens (n = 4). Statistical significance across groups was assessed using one-way ANOVA and Tukey’s posttest. Significance levels are indicated as follows: **P* < 0.05, ****P* < 0.001.

**Figure** **S22.** Representative flow cytometric plots showing the percentages of mature dendritic cells (DCs) (A), M2 macrophages (B), CD4^+^ T cells (C), CD8^+^Granzyme B^+^ (GrB^+^) T cells (D), and regulatory T cells (Tregs) (E) in the primary TDLNs of mice with different treatments.

**Figure** **S23.** Representative flow cytometric plots showing the percentages of (A) mature dendritic cells (DCs), (B) CD4^+^ T cells, and (C) CD8^+^ T cells in the spleen tissues of mice with various treatments.

**Figure** **S24**. Representative flow cytometric plots and corresponding quantitative analysis showing the percentages of M1 macrophages in the spleen tissues of mice with different treatments (n=3). Statistical significance across groups was assessed using one-way ANOVA and Tukey’s posttest. Significance levels are indicated as follows: **P* < 0.05.

**Figure** **S25.** Representative flow cytometric plots showing the percentages of (A) mature dendritic cells (DCs), and (B) M2 macrophages in the distant TDLNs of mice with different treatments.

**Figure** **S26.** Representative flow cytometric plots and corresponding quantitative analysis showing the percentages of regulatory T cells (Tregs) in the distant TDLNs of mice with different treatments (n=3). Statistical significance across groups was assessed using one-way ANOVA and Tukey’s posttest. Significance levels are indicated as follows: ***P* < 0.01.

**Figure S27.** Representative flow cytometric plots and corresponding quantitative analysis showing the percentages of central memory T cells (T_CM_) in the distant TDLNs of mice with different treatments (n=4). Statistical significance across groups was assessed using one-way ANOVA and Tukey’s posttest. Significance levels are indicated as follows: ***P* < 0.01.

**Figure** **S28.** Representative flow cytometric plots showing the percentages of mature DCs (A), and M1 macrophages (B) in the distant tumors of mice with different treatments.

**Figure** **S29.** The luminescence quantitative analysis was based on the IVIS images of lung tissues depicted in Figure 9C (n = 4–5). Data are presented as mean ± SD. Statistical significance was determined using one-way ANOVA with Tukey’s post-hoc test for multiple group comparisons. Significance levels are indicated as follows: ***P* < 0.01.

**Figure** **S30.** Representative flow cytometric plots showing the percentages of mature dendritic cells (DCs) (A), M2 macrophages (B), and CD8^+^ T cells (C) in the TDLNs of mice with different treatments. (D) Representative flow cytometric plots and corresponding quantitative analysis showing the percentages of CD4^+^ T cells in the TDLNs of mice with different treatments (n=3-4). Representative flow cytometric plots showing the percentages of Tregs (E) in the TDLNs of mice with different treatments. Statistical significance across groups was assessed using one-way ANOVA and Tukey’s posttest. Significance levels are indicated as follows: **P* < 0.05, ***P* < 0.01.

**Figure** **S31.** Representative flow cytometric plots showing the percentages of (A) mature dendritic cells (DCs), (B) M1 macrophages, (C) CD8^+^ T cells, (D) central memory T cells (T_CM_), and (E) effector memory T cells (T_EM_) in the spleens of mice with different treatments.

**Figure** **S32.** Representative flow cytometric plots and corresponding quantitative analysis showing the percentages of CD4^+^ T cells in the spleens of mice with different treatments (n=3). Statistical significance across groups was assessed using one-way ANOVA and Tukey’s posttest. Significance levels are indicated as follows: **P* < 0.05.

**Figure** **S33.** Representative flow cytometric plots showing the percentages of M1 macrophages (A), CD8^+^ T cells (B), and T_CM_ (C) in the lungs of mice with different treatments.

**Methods**

**Materials**

Selenium (99.9% metals basis, CAS: 7782-49-2), Bi(NO_3_)_3_•5H_2_O (AR, 99.0%, CAS:10035-06-0), PVP (MW：10000), 1-tetradecanol (AR, 98.0%, CAS:112-72-1), hyaluronic acid (97%, 30-45 KDa, CAS: 9004-61-9), and agar powder (Biochemical reagent, Cat. A800730-250 g) were purchased from Shanghai Macklin Biochemical Co. Ltd. (Shanghai, China). NaBH_4_ (98%, CAS:16940-66-2), Triton-X100 (Biochemical grade), NaOH (ACS, 97%, CAS:1310-73-2), 3,3’,5,5’-tetramethyl-benzidine (TMB, >99%, HPLC, CAS:54827-17-7), 1-ethyl-3-(3-dimethylaminopropyl)carbodiimide(EDC, 98%, CAS:25952-53-8), and ICG (75%, CAS: 3599-32-4) were ordered from Aladdin (Shanghai, China). RBITC (Cat. 283924, Powder), 2’,7’-dichlorodihydrofluorescein diacetate (DCFH-DA, Cat. 4091990), yeast (YSC2-500G), and bovine serum albumin (BSA, SRE0096) were obtained from Sigma-Aldrich (Shanghai, China). CuCl_2_•2H_2_O (AR, 99%) was purchased from Damao Chemical Reagent Factory (Tianjin, China). H_2_O_2_ (30%, analytical reagent) and glucose (analytical reagent, CAS: 50-99-7) were ordered from the Guangzhou Chemical Reagent Factory. Ethanol absolute (AR, CAS:64-17-5) was purchased from Guangdong Guanghua Sci-Tech Co., Ltd. SCH442416 (Cat. S10832) was ordered from Tideradar Beijing Technology Co., LTD. DSPE-PEG-NH_2_ (95% MW 2000 Da, Cat. PS2-NE1-2K-1 g) was purchased from Ponsure biotechnology (Shanghai, China). DSPE-PEG-NHS (95% MW2000, Cat. P006005-2K) was ordered from Shanghai ToYongBio Tech. Inc. 4% paraformaldehyde (Cat. BL539A) was obtained from Biosharp Life Sciences Co., Ltd. Yeast extract powder (Cat. S24010-250 g), and tryptone (Cat. S31316-500 g) were purchased from Shanghai Yuanye Bio-Technology Co., Ltd. Lyso-Tracker green (Cat. C1047S), Calcein-AM/PI cell viability assay kit (Cat. C2015M), ATP assay kit (Cat. S0026), DNA damage detection kit (γ-H2AX immunofluorescence assay, Cat. C2035S), one-step TUNEL cell apoptosis detection kit (Cat: C1088), and GSH/GSSG detection kit (Cat. S1003), were purchased from Beyotime Biotechnology (China). Dulbecco’s phosphate-buffered saline (DPBS), Annexin V-FITC/PI apoptosis detection kit (Cat. 40302ES60), collagenase IV (Cat. 40510ES60), hyaluronidase (Cat. 20426ES60), Super ECL detection reagent (Cat. 36208ES60) and D-luciferin potassium salt (Cat. 40902ES03) were obtained from YEASEN Biotechnology Co. Ltd. (Shanghai, China). Alamar Blue^TM^ cell viability reagent (Cat. DAL1100), DAPI (Cat. D1306), Hoechst 33342 (R37605), Foxp3/Transcription factor staining buffer set (Cat. 00552300, eBioscience™), and intracellular fixation & permeabilization buffer set (Cat. 88882400, eBioscience™) were purchased from Invitrogen Co. Ltd. Anti-calreticulin antibody [EPR3924]-ER marker (ab92516), recombinant Alexa Fluor® 488 Anti-Ki67 antibody [SP6] (Cat. ab281847), goat anti-rabbit IgG H&L (Alexa Fluor® 488) (Cat. ab150077), recombinant anti-CD8 alpha antibody [EPR21769] (ab217344), anti-adenosine receptor A2a antibody (Cat. ab3461), recombinant anti-CD4 antibodies [EPR19514] (ab183685) were purchased from Abcam Co. Ltd. (Shanghai, China). HMGB1 antibody (Cat. 3935S), anti-rabbit IgG, and HRP-linked antibody (Cat. 7074P2) were ordered from Cell Signaling Technology Inc. (Beverly, MA, USA). Anti -FOXP3 rabbit pAb (Cat. GB11093) was purchased from Wuhan Servicebio Technology Co. Ltd. HMGB1 ELISA kit (Cat. SP14752) was ordered from Wuhan Saipei Biotechnology Co., Ltd. Anti-mouse PD-L1 (B7-H1) (Cat. BP0101) was ordered from Bioxcell. Red blood cell lysis buffer (Cat. R1010) and RIPA tissue/cell lysates (Cat. R0020) were purchased from Solarbio Life Sciences Co. Ltd. (Beijing, China). 5’-N-Ethylcarboxamidoadenosine (NECA) (Cat. B5164) was purchased from APExBIO. 10×TBST (Cat. E175-01) was purchased from GenStar. Leukocyte activation cocktail, with BD GolgiPlug^TM^ (Cat. 550583) was ordered from BD Biosciences.

DNase I (Cat. 10104159001) was purchased from Roche. CD16/CD32 monoclonal antibody (93) (Cat. 14016182), CD45 monoclonal antibody (30F11) (PE-Cyanine7, Cat. 25045182), Fixable viability dye eFluor™ 780 (Cat. 65086518), CD3e monoclonal antibody (145-2C11) (FITC, Cat. 11003185), CD62L (L-Selectin) monoclonal antibody (MEL-14) (APC, Cat. 17062182), CD4 monoclonal antibody (GK1.5) (eFluor™ 450, Cat. 48004182), CD44 monoclonal antibody (IM7) (PE, Cat. 12044181), FOXP3 monoclonal antibody (FJK-16s) (PE, Cat. 12577382), CD8a monoclonal antibody (53-6.7) (eFluor™ 506, Cat. 69008182), CD206 (MMR) monoclonal antibody (MR6F3) (PE, Cat. 12206182), IFN gamma monoclonal antibody (XMG1.2) (APC, Cat. 17731182), CD86 (B7-2) monoclonal antibody (GL1) (APC, Cat. 17086282), CD80 (B7-1) monoclonal antibody (16-10A1) (PE, Cat. 12080182), CD11c monoclonal antibody (N418) (FITC, Cat. 11011485), CD11b monoclonal antibody (M1/70) (FITC, Cat. 11011285), and F4/80 monoclonal antibody (BM8) (eFluor™ 450, Cat. 48480182) were ordered from eBioscience (San Diego, CA, USA).

**Instruments and Software**

Hydrodynamic size and zeta potential were determined by dynamic light scattering (DLS) and electrophoretic light scattering on a Zetasizer Nano ZS (Malvern Panalytical). UV–vis absorbance spectra were recorded on a JASCO P-2000 spectrophotometer. Transmission electron microscopy (TEM) and high-resolution TEM (HRTEM) images were acquired on an FEI Tecnai G2 F20 S-TWIN. Flow cytometry data were analyzed using FlowJo v10.8.1, while statistical analyses were performed with GraphPad Prism 10. Infrared thermal imaging data were processed using AnalyzIR v4.3.3.108, relative quantification of fluorescence images was done with ImageJ v1.53t, and in vivo bioluminescence data were analyzed using Living Image v4.3.1.16427.
